# Membrane adsorbers with ultrahigh metal-organic framework loading for high flux separations

**DOI:** 10.1038/s41467-019-12114-8

**Published:** 2019-09-16

**Authors:** Hang Wang, Shuang Zhao, Yi Liu, Ruxin Yao, Xiaoqi Wang, Yuhua Cao, Dou Ma, Mingchu Zou, Anyuan Cao, Xiao Feng, Bo Wang

**Affiliations:** 1 Beijing Key Laboratory of Photoelectronic/Electrophotonic Conversion Materials, Key Laboratory of Cluster Science, Ministry of Education, School of Chemistry and Chemical Engineering, Beijing Institute of Technology, Beijing, 100081 P. R. China; 2 PetroChina Research Institute of Petroleum Exploration & Development, Beijing, 100083 P. R. China; 3 Department of Materials Science and Engineering College of Engineering, Peking University, Beijing, 100871 P. R. China; 4 Department of Chemistry, Tsinghua University, Beijing, 100084 P. R. China

**Keywords:** Metal-organic frameworks

## Abstract

Metal-organic frameworks (MOFs) with high porosity and designable functionality make it possible to access the merits of high permeability and selectivity. However, scalable fabrication methods to produce mixed matrix membranes (MMMs) with good flexibility and ultrahigh MOF loading are urgently needed yet largely unmet. Herein, we report a thermally induced phase separation-hot pressing (TIPS-HoP) strategy to roll-to-roll produce 10 distinct MOF-membranes (loadings up to 86 wt%). Ultrahigh-molecular-weight polyethylene interweaving the MOF particles contributes to their mechanical strength. Rejections (99%) of organic dyes with a water flux of 125.7 L m^–2^ h^–1^ bar^–1^ under cross-flow filtration mode. The micron-sized channels between the MOF particles translate into fast water permeation, while the porous MOFs reject solutes through rapid adsorption. This strategy paves ways for developing high-performance membrane adsorbers for crucial separation processes. As a proof-of-concept, the abilities of the membrane adsorbers for separating racemates and proteins have been demonstrated.

## Abstract (web summary)

Mixed matrix membranes have shown great promise for separation applications, but low filler loading typically leads to low selectivity. Here the authors use a thermally induced phase separation-hot-pressing strategy to fabricate 10 distinct metal-organic framework-based membrane adsorbers with up to 86 wt% MOF-loading.

## Introduction

Separation is of vital importance in modern society^1^. Water purification, as one of the most important separation processes, is critical to address the growing global concerns on water scarcity and quality^2,3^. While in pharmaceutical and biological industries, efficient chiral and protein separations are also essential in the production of optically active drugs and study of their biological activities^4,5^. Metal-organic frameworks (MOFs) are crystalline materials assembled by metal ions with organic struts^6–8^. In addition to their rich chemistry, they possess ordered open channels, large porosities, predictable pore size, and adjustable chemical environment, offering great opportunities for separation applications^9–12^. Despite the fact that MOF crystalline powders have the ability to separate dye molecules based on adsorption, they are brittle in nature and can easily break down into fine powders. In industrial applications, these tiny particles will clog the pipes during separation and severe material loss will inevitably occur when flushing with fluid^13^. In addition, membrane separation that does not rely on heat requires much lower energy and less space compared with conventional industrial separation methods^1,14–16^. Particularly, membrane adsorbers are able to sufficiently increase the flow rate through anchoring functional groups onto the porous membranes (e.g., resins). However, they can only treat solutions with very low concentrations due to limited porosity and adsorption ability. Therefore, incorporation of porous and functional MOFs into membrane adsorbers with ultrahigh loading is highly desired.

Various approaches have been developed to improve their processabilities, for examples, growing or depositing MOF crystals on a porous substrate via in situ chemical/electrochemical growth^17^, layer deposition^18^, and step-by-step liquid-phase epitaxial growth^19^; blending MOFs with polymers to yield mixed matrix membranes (MMMs)^20–23^. Among them, some pioneering works have shown liquid-phase separation behaviors. Based on separation mechanisms, MOFs usually act as molecule-sieving channels in the continuous MOF film and serve as fillers and/or adsorbers in MMMs^20,24^. However, there still remain unsurmountable challenges. Large-area and continuous defect-free MOF separation layers are difficult to fabricate; MOF membrane adsorbers that can be used for treating a small amount of wastewater with low pollutant concentration suffer from pulverization problem, having limited application in practice^25–27^; for MOF-based MMMs, high MOF loadings lead to phase segregation with increased fragility and bring the difficulties in the producing of large-area membranes without cracks, while low loadings usually result in low specific adsorption sites and/or separation channels/sites and consequently cause low selectivity^22,28^.

Nonsolvent-induced phase separation (NIPS) and thermally induced phase separation (TIPS) are well-established methods in the industry for production of polymeric microfiltration and ultrafiltration membranes with reduced fabrication cost^29,30^. However, membranes prepared by the NIPS or TIPS method usually reject large macromolecules and particulate matters (i.e., proteins, suspended solids, bacteria, virus, and colloids), but fail to precisely separate small organic molecules^31^. Further reduction in the pore size is required to sieve molecules yet inevitably leads to significantly lower water flux, accompanied with a huge energy consumption^32^. Moreover, traditional membranes usually lack functionalizability and tunability at the molecular level, resulting in difficulties for separating enantiomers and proteins with similar molecular size. Incorporating MOFs with designed functionalities and adsorption sites into membranes prepared by NIPS or TIPS method may provide an ideal complement to current microfiltration and ultrafiltration membrane technology. NIPS method has been utilized to prepare MMMs with MOF loadings <5%^33–35^, where the MOFs serve as fillers to increase the water flux. However, these MMMs still suffer from the tradeoff between the selectivity and permeance, for example, MOF-based nanofiltration membrane for small-molecule removal (i.e., dyes and heavy metal ions) normally possess poor water permeance (< 10 L m^–2^ h^–1^ bar^–1^)^16^. It is supposed that increasing MOF loading can benefit the volume percentage of the pores with desired pore size and adsorption sites to trap solute and can also promote water permeation through the open channels, and thus offering opportunities to break the tradeoff dilemma. Since the mechanical strength of the MMMs is dominated by the polymeric materials, we hypothesized that polymers with higher-molecular weight (i.e., UHMWPE) may impart the corresponding MMMs with good mechanical strength without sacrificing the MOF loading. However, the poor solubility of the polymers with high-molecular weight cannot fulfill the prerequisite for the NIPS method to afford membranes.

**Fig. 1:**
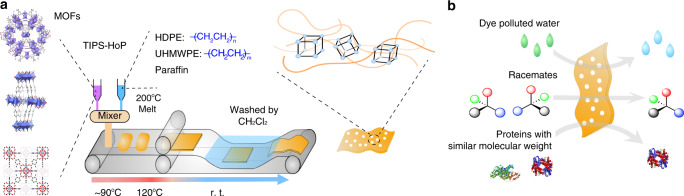
MOF PE MMMs prepared by TIPS-HoP. **a** Schematic of the MOF PE MMMs fabrication process using the TIPS-HoP method. **b** Schematic illustration of the MOF PE MMMs for dye, racemates, and protein separations

Therefore, we turned to the TIPS process that requires the thermal plastic melting rather than the solubility of polymers to incorporate high-molecular weight with high MOF loadings. We judiciously propose a combination of TIPS and hot-pressing method (TIPS-HoP) for preparing large-area ultrahigh MOF loading membranes with strong thermal and chemical robustness, as well as good mechanical strength. In the TIPS-HoP procedure, MOF nanocrystals are first dispersed and suspended in the melt of high-density polyethylene (HDPE), ultrahigh-molecular-weight polyethylene (UHMWPE) and paraffin at 200 °C, and then the resulting mixture is placed onto the belt followed by roll-to-roll hot pressing to form membrane at 120 °C and removing of paraffin by soaking in the organic solvent. Ten MOF PE MMMs with high flexibility are fabricated through this TIPS-HoP strategy, and up to 86% MOF loading can be achieved irrespective of the kind of MOFs (Fig. 1a). Ultrahigh-molecular weight PE contributes to the linking up of MOF particles to produce flexibility, while micron-sized channels between the MOF particles and intrinsic nanosized cavities in the MOFs endow them with the possibilities to serve as separation membrane adsorbers with both high selectivity and high flux. We intentionally select NH_2_-UiO-66 PE MMM, MIL-100(Cr) PE MMM, Zn-BLD PE MMM, and NH_2_-UiO-66-MIL-100(Cr) PE MMM with specific adsorption sites (basic side chains in NH_2_-UiO-66^36^, open metal sites in MIL-100(Cr)^37^, and chiral ligands in Zn-BLD^38^), respectively, to perform dye, racemate, and protein separation tests (Fig. 1b). Under similar pollutant concentration, cross-flow filtration mode, and regeneration process, these MMM adsorbers exhibit superior performance for separating specific molecules comparing with state-of-the-art membranes reported to date in terms of permeance and selectivity. Since the TIPS method is a well-established technique, our TIPS-HoP strategy is promising for continuous roll-to-roll fabrication of high MOF loading membrane adsorbers with preferable functions for chemical and biological separations.

## Results

### The fabrication of MOF PE MMMs

The combinations of HDPE and paraffin, as well as UHMWPE and paraffin, are both classical formulae in the TIPS technology, where paraffin acts as a flowable agent to reduce viscosity^39^. It is found that either brittle or impermeable membranes are formed by using these formulae, due to too low or too high melt viscosity of HDPE and UHMWPE, respectively. Thus, only proper ratio of HDPE, UHMWPE, and paraffin is able to make them amenable to conventional melt and further shaping processes. Subsequently, we chose the hot-pressing method to fabricate membranes by shaping the bodies formed during the cooling of the melt mixtures.

**Fig. 2:**
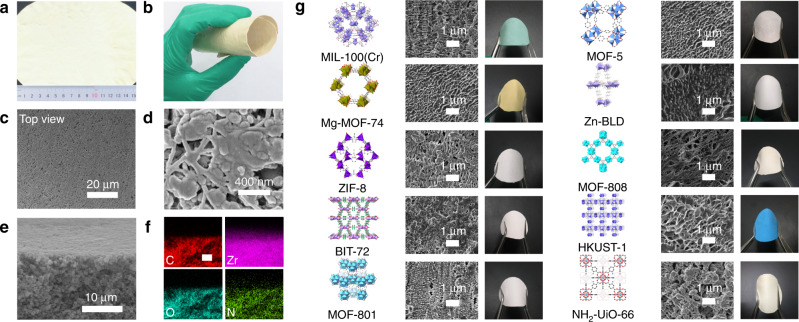
Ten representative MOF PE MMMs. **a**, **b** Photographs of NH_2_-UiO-66 PE MMM-86% with an area of 15 cm × 13 cm. **c**, **d** Top view SEM images of NH_2_-UiO-66 PE MMM-86% (scale bar, 20 μm for **c** and 400 nm for **d**). **e**, **f** Cross-section SEM image of the NH_2_-UiO-66 PE MMM-86% and corresponding spatial distribution of C, N, O, and Zr (scale bar, 10 μm). **g** Chemical structures of MOF crystals and SEM images and photographs of the corresponding PE MMMs with 86 wt% MOF loading (scale bar, 1 μm)

A typical and optimized procedure for fabricating MOF PE MMM-*w*% (*w*% denoted to the weight percentage of MOFs) by the TIPS-HoP method is performed as follows: first, MOF crystals were blended with HDPE (M_w_ > 40,000, melt index = 2.2 g per 10 min), UHMWPE (M_w_ > 1,500,000), and paraffin via vigorous stirring at 200 °C, at which temperature HDPE and UHMWPE are melt and homogeneously mixed with liquid paraffin; second, the mixture was placed onto the belt at ambient atmosphere accompanied with a temperature reducing to ~90 °C and a solidification to form bulk substance with high softness; third, the resultant bulk mixture was shaped into membranes via a roll-to-roll hot-pressing at 120 °C with a speed of 40 rpm; finally, the MOF PE MMM with plentiful macro-porosities and micro-porosities was obtained by soaking it in CH_2_Cl_2_ to completely wash out paraffin. The MOF loadings calculated from the TGA analyses are consistent with the theoretical values (Supplementary Fig. 1, Supplementary Table 1), proving that the paraffin is completely removed from the MMMs. Flexible MOF PE MMMs with different sizes are displayed (Fig. 2a, b; Supplementary Fig. 2), and this roll-to-roll production can be easily scaled up by retrofitting in current industrial facilities and processes.

### Characterizations of MOF PE MMMs

Taking NH_2_-UiO-66 PE MMM as an example, we adjusted the MOF loading amounts from 28%, 54%, 61%, 70%, 86% to 92%. All of the obtained NH_2_-UiO-66 PE MMMs adopt acceptable mechanical strength and can be used for the permeation tests, and most of them are highly flexible except for the membrane with 92% NH_2_-UiO-66 loading. The stress–strain curves of NH_2_-UiO-66 PE MMMs with different MOF loadings were measured (Supplementary Fig. 3, Supplementary Table 2). The mechanical performance indeed decreases with the increasing of MOF loading, but NH_2_-UiO-66 PE MMM-86% still possesses the flexibility and can bear bending without the formation of cracks. In comparison, we first attempted to prepare MMMs with an 86% MOF loading via NIPS approach by using polyvinylidene fluoride (PVDF, M_w_ = 430,000) and polyacrylonitrile (PAN, M_w_ = 150,000) as the matrix. The obtained membranes, NH_2_-UiO-66 PVDF MMM-86% and NH_2_-UiO-66 PAN MMM-86%, are extremely fragile and show plenty of pinholes and cracks visible to the naked eye (Supplementary Figs. 3, 4).

As shown in the top views of field-emission scanning electron microscope (FE-SEM) images, MMMs with <70% loading exhibit flake texture morphology, while structured curly strands are formed in NH_2_-UiO-66 PE MMM-70% and obvious MOF particle aggregates interwoven by long-chain polymers are observed in 86 and 92% loading MMMs (Fig. 2c, d; Supplementary Fig. 5). Unlike the significantly distinct morphologies in the top surfaces, the cross-section images reveal that NH_2_-UiO-66 PE MMM with different loadings adopts similar morphology except for the density of the polymer strands (Supplementary Fig. 6). FE-SEM images demonstrate that all the obtained NH_2_-UiO-66 PE MMMs are symmetrical membranes and possess massive porosities and micro-sized channels. Although MOF particle aggregates can be observed, elemental mappings reveal their distributions are almost uniform throughout the membranes (Fig. 2e, f). X-ray fluorescence image of NH_2_-UiO-66 PE MMM-86% with a diameter of 6.5 cm also indicate that the Zr element is evenly distributed in the large area (Supplementary Fig. 7).

The powder X-ray diffraction (PXRD) patterns of the NH_2_-UiO-66 PE MMM with different loadings are consistent with that of pure NH_2_-UiO-66 single crystals and simulation, indicating that their underlying topology remains intact after TIPS-HoP processing (Supplementary Fig. 8). Fourier-transform infrared attenuated total reflectance (FTIR-ATR) spectra indicate the chemical bonds are retained for each component. Further chemical stability tests were conducted by immersing NH_2_-UiO-66 PE MMM-86% in methanol, basic (pH = 10) and acid (pH = 3) aqueous solutions, and the results show that the morphology, as well as the PXRD patterns and FTIR-ATR spectra of the NH_2_-UiO-66 PE MMMs remain unchanged (Supplementary Figs. 9, 10). N_2_ sorption isotherms (Supplementary Fig. 11) were measured to determine the porosity of the NH_2_-UiO-66 PE MMMs. The Brunauer–Emmett–Teller surface areas are calculated to be 0, 50, 212, 649, 746, and 839 m^2 ^g^–1^ for the NH_2_-UiO-66 PE MMM with 28%, 54%, 61%, 70%, 86, and 92% loading, respectively, revealing the majority of the intrinsic porosities of the MOFs are fully accessible. Since NH_2_-UiO-66 has a BET surface area of 1035 m^2 ^g^–1^ and PE is nonporous, it should be noted that a small number of pores in the MOFs are blocked by the polymers in the NH_2_-UiO-66 PE MMMs considering the weight contributions of individual components (Supplementary Table 3). These results and morphology observations indicate that the MOF crystals are interwoven by ultrahigh-molecular-weight PE, ensuring efficient linking up of MOF particles and avoiding pulverization even under ultrahigh MOF loadings.

To demonstrate the versatility of the TIPS-HoP method to fabricate membranes with ultrahigh MOF loadings, we applied it to prepare a series of representative MOF PE MMMs, including ZIF-8, MOF-5, MOF-801, MOF-808, HKUST-1, MIL-100(Cr), BIT-72, Mg-MOF-74, and Zn-BLD PE MMM-86%. All of these MMMs show good flexibility and mechanical strength, and adopt similar micromorphology to NH_2_-UiO-66 PE MMM-86% (Fig. 2g). The PXRD and N_2_ sorption isotherm measurements indicate that the crystal structures and most porosities of the MOFs are retained in the MMMs (Supplementary Figs. 12, 13). In addition to the hierarchical porous structure, including both macro-pores formed between the interwoven MOF particles and micropores or mesopores inside the MOFs, the rich chemistry in MOFs imparts the MMMs with the abilities for further selective and efficient separation applications.

### Dyes removal performance

**Fig. 3:**
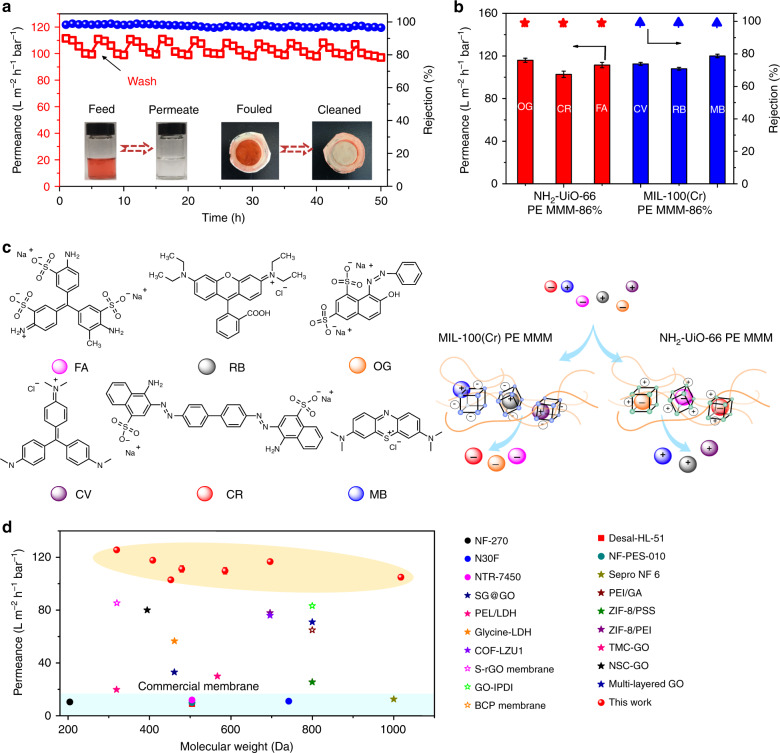
MOF PE MMMs for dyes removal. **a** Antifouling performance and long-term stability of NH_2_-UiO-66 PE MMM-86% for the CR removal. The membrane was activated by washing with saturated NaNO_3_ methanol solution in the cross-flow system during the cycles. **b** The separation performance of NH_2_-UiO-66 PE MMM-86% and MIL-100(Cr) PE MMM-86% for different dyes (concentration, 100 ppm; applied pressure is 0.2 MPa). **c** Schematic illustrations of the mechanism for dyes removal by MOF PE MMMs. **d** A performance comparison of NH_2_-UiO-66-MIL-100(Cr) PE MMM-86% with commercial and literature reported membranes. Error bars in **b**, **d** indicate the standard deviation of three independent samples

We first chose NH_2_-UiO-66 PE MMMs to examine their performance for dye removal. The filtration experiments were performed in a cross-flow system (flow rate is 70 L h^–1^) (Supplementary Fig. 14). In the tests for the removal of Congo red (CR) from its aqueous solution (100 ppm) (Supplementary Fig. 15), the water cannot permeate the MMMs until the MOF loading reaches 54%. The water permeance is remarkably improved with the further increase of MOF loadings and achieves 126.9 L m^–2^ h^–1^ bar^–1^ for the NH_2_-UiO-66 PE MMM-92%, along with the CR rejections all higher than 99.0%. Considering the mechanical behavior and separation performance, we used NH_2_-UiO-66 PE MMM-86% for further testing. During the long-term measurement, the permeance only declines 10% after 5 h of nonstop separation (Fig. 3a), and this outstanding antifouling performance is highly valuable for practical application. The contaminant accumulated on the surface and inside of the membrane adsorbers can be removed, and the permeance of the fouled NH_2_-UiO-66 PE MMM-86% can be easily recovered by washing with NaNO_3_ methanol solution for 5 min in the cross-flow system with a flux recovery ratio (FRR) as high as 97% (Fig. 3a). After ten CR removal circles (5 h for each cycle), the water flux and CR rejection of NH_2_-UiO-66 PE MMM-86% do not show any obvious deterioration.

The BET surface area of the NH_2_-UiO-66 PE MMM-86% adsorber after 5-h CR removal test decreases from 746 to 253 m^2 ^g^–1^ and can be easily regenerated to 80% of its original porosity after washing (Supplementary Fig. 16). The pore size distributions (Supplementary Fig. 16) reveal that the micropores inside MOFs are partially filled by dye molecules. The SEM images show that the dye adsorption also occurred on the surface (Supplementary Fig. 17). To further confirm the function of the hierarchical porous structure for CR removal, we fabricated nonporous Al_2_O_3_- and ZnO-mixed membranes (the particle sizes of ZnO and Al_2_O_3_ are 200 and 50 nm, respectively) using identical TIPS-HoP method as comparisons (Supplementary Fig. 18). These two membranes exhibit extremely low water flux and almost no CR rejection ability. Then we carefully tested the sorption ability of NH_2_-UiO-66 powder for CR. The adsorption kinetic data fitted well by the pseudo-second-order equation (*R*^2 ^> 99.6%) with a second-order rate constant of 10 mg mg^–1^ min^–1^, and the capacity is calculated to be 697.7 mg g^–1^ by fitting the adsorption isotherm with Langmuir model (Supplementary Fig. 19, Supplementary Tables 4, 5). These results demonstrate the functionalized and enriched pore structure of NH_2_-UiO-66 adsorber contributes to both water flux and specific dye capture.

It is reported that NH_2_-UiO-66 powders can selectively adsorb negatively charged dye molecules through electrostatic attraction^28,40,41^. To evaluate the surface charge of the MMMs, the zeta potential analyses were conducted (Supplementary Fig. 20). It is observed that the NH_2_-UiO-66 PE MMM-86% adopts a positive potential in the aqueous solution (pH = 7), which is mainly originated from the protonation of the amino groups anchored to the inner pore surface of the MOF^40,42^. Then we tested the removal ability of NH_2_-UiO-66 PE MMM-86% adsorber for another five dye molecules, including crystal violet (CV), rhodamine B (RB), methylene blue (MB), fuchsine acid (FA), and orange G (OG). In consistent with adsorption behavior of powder samples, the NH_2_-UiO-66 PE MMM-86% can selectively reject FA (99.1% rejection, 111.4 L m^–2^ h^–1^ bar^–1^ permeance) and OG (99.0% rejection, 115.9 L m^–2^ h^–1^ bar^–1^ permeance) and allow CV, RB, and MB to permeate (Fig. 3b; Supplementary Fig. 21, Table 6).

To separate positively charged dyes, we utilized a negatively charged membrane (MIL-100(Cr) PE MMM-86%) (Supplementary Fig. 20). As expected, MIL-100(Cr) PE MMM-86% adsorber is able to remove CV (99.0% rejection, 112.5 L m^–2^ h^–1^ bar^–1^ permeance), RB (99.2% rejection, 108 L m^–2^ h^–1^ bar^–1^ permeance) and MB (99.2% rejection, 120 L m^–2^ h^–1^ bar^–1^ permeance) and allows CR, FA, OG to pass through (Fig. 3b; Supplementary Fig. 21, Table 6). We also prepared an 86% loading activated carbon membrane adsorber (Supplementary Fig. 18). Although the activated carbon is highly porous with a surface area of 1410 m^2 ^g^–1^, the resultant membrane failed to remove any of these six dyes due to its neutral nature.

The above results reveal that the electrostatic attraction governs the selectivity to the dyes, and the hierarchical porous structure contributes to both high water flux and dyes removal (Fig. 3c). The fast dye adsorption ability of the MMM adsorbers was further exhibited by adding a drop of dye solution onto their surface. The photos show that the color quickly fades within 30 s for the MMM adsorbers, whereas no changes are observed for the ZnO, Al_2_O_3_, and activated carbon membranes (Supplementary Fig. 22).

Then we prepared NH_2_-UiO-66-MIL-100(Cr) PE MMM-86% (*w*(NH_2_-UiO-66)/*w*(MIL-100(Cr)) = 1:1). It can sufficiently reject (> 99.0%) all the selected target pollutants while keeping the water flux higher than 100 L m^–2^ h^–1^ bar^−1^ (Fig. 3d; Supplementary Fig. 23). It is worth noting that not only charged dyes but also uncharged dye (rose bengal) can be efficiently removed by this mixed PE MMM adsorber (Supplementary Table 7). For separating specific dye molecules with similar dye concentration and regeneration process, to the best of our knowledge, its water flux outperforms all state-of-the-art commercial and literature reported membranes (Fig. 3d; Supplementary Table 7). It should be noted that only molecules with suitable sizes that can enter the pores are able to be separated by these MMM adsorbers through adsorption, but it still can serve as good complement to current microfiltration and ultrafiltration membrane technology in terms of high permeance and designability.

To further demonstrate the advantages of these membranes, we packed MOF powders on substrates or in columns, where the MOF behaves as a adsorber. Since the interaction between the MOF and the substrate is weak and the MOF particles can easily fall off without adhesion of additional polymeric binders, leading to the difficulties in performing separation process in cross-flow filtration mode. We filled columns by using NH_2_-UiO-66 powders (0.1 g, the same MOF amount in a 3.14 cm^2^ NH_2_-UiO-66 PE MMM-86%) for fuchsine acid (FA) separation (100 ppm). In the column with the same area (3.14 cm^2^) of MMM used for separation (Supplementary Fig. 24), the flux is high but cannot reject dye molecule. In the column with a diameter of 6 mm, the resistance is too high to allow the permeation of the solvent (Supplementary Fig. 24) even under a pressure of 0.1 MPa. The column is clogged by the nanosized MOF particles. When we packed the same amount of 0.086 g NH_2_-UiO-66 mixed with 0.0112 g HDPE and 0.0028 g UHMWPE to fill a column (i.d. 6 mm) to perform fuchsine acid (FA) separation (100 ppm) under 0.1 MPa, the result is the same with pure MOF powder due to the very low proportion of polymer. Then we tried to use quartz sand to disperse MOF particles (0.1 g, same amount of an MMM with an area of 3.14 cm^2^) under atmospheric pressure, the rejection for FA (100 ppm) was calculated to be only 47.7% (Supplementary Fig. 25).

### Chiral and protein separation performance

The rich MOF chemistry offers a toolbox to expand the application realm of the MMM adsorbers, and most importantly, the high MOF loading achieved by the TIPS-HoP method impart them with excellent performance. As a proof-of-concept, we further utilized Zn-BLD PE MMM-86% and MIL-100(Cr) PE MMM-86% to separate enantiomers (R-/S-methyl phenyl sulfoxide (R-/S-MPS)) and proteins with similar molecular size (BSA (14 × 3.8 × 3.8 nm, 65 kDa) and BHb (6.4 × 5.5 × 5 nm, 66 kDa)) under dead-end filtration mode, respectively.

Zn-BLD is a chiral MOF and it can be used for separating R-/S-MPS through selective adsorption^38,43^, therefore, we chose it to prepare Zn-BLD PE MMM-86% adsorber and perform enantiomers (concentration, 0.1 mg mL^–1^) separation test. We collected the permeate after 15-min dynamic separation. The results show that the enantiomeric excess (ee%) value is ~74% with acetonitrile permeance of 85 L m^–2^ h^–1^ bar^–1^ (Supplementary Fig. 26). The ee% value reduces to 47.8% from 15 to 30 min, and will be further decreased with prolonged time. However, both ee% value and solvent permeance performance can be recovered by washing, and also remained cross five cycles (Supplementary Figs. 26–28). The enantiomer permeance of R-MPS is calculated to be 11.5 ± 0.5 mmol m^–2^ h^–1^ (Supplementary Fig. 29). This ee% value is among the best for the membrane-based chiral separations, and enantiomer permeance is hardly achieved by the reported membranes with similar separation ee% value^44–46^ (Supplementary Table 8). The ee% value by using NH_2_-UiO-66 PE MMM-86% for R-/S-MPS separation is close to zero, revealing that the membrane without chirality cannot be used to separate enantiomers (Supplementary Fig. 30).

**Fig. 4:**
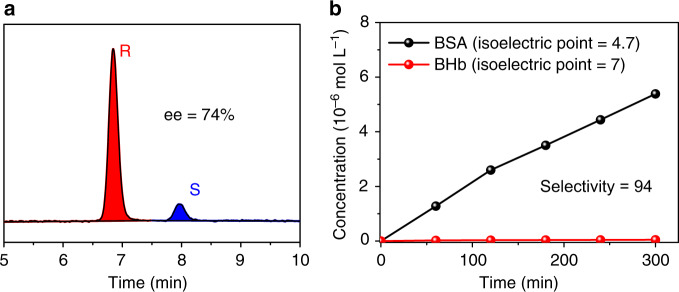
MOF PE MMM adsorbers for chiral resolution and proteins separation. **a** High-performance liquid chromatogram of chiral MPS permeate after filtration by Zn-BLD PE MMM-86%. **b** Proteins (BSA and BHb) concentration profiles in the permeate filtrated by MIL-100(Cr) PE MMM-86% versus time

BSA and BHb have similar size but different isoelectric point, and it is reported that they can be separated through electrostatic attraction^47–49^. We performed the protein separation under a pH of 4.7, at which condition the BHb becomes positively charged and BSA is neutral^47^. The selective factor of 94 is achieved by using MIL-100(Cr) PE MMM adsorber with negative surface charge, which is the highest value compared with the literature reported membranes (Fig. 4b; Supplementary Table 9). Since the molecular sizes of the proteins are larger than the pore size of MIL-100(Cr), we attributed the separation behavior is mainly originated from the electrostatic attraction.

## Discussion

The MOF membrane adsorber separation process, unlike traditional nanofiltration, is mostly based on adsorption mechanism. MOF PE MMM adsorbers require periodical desorption and cleaning to recover the capability for separation of small molecules. However, it is worth noting that the membrane cleaning is indeed a standard routine operation procedure in many separation processes, especially nanofiltration and reverse osmosis, since the fouling would lead to a sharp decrease of permeate flux. The antifouling and recovery performance of MOF PE MMM adsorbers are actually superior to those of the reported filtration membranes (Supplementary Table 10). Importantly, no additional energy input is required during the MOF membrane adsorber separation process. Specifically, we performed the dye separation experiments in comparable conditions commonly used in microfiltration and ultrafiltration (i.e., cross-flow filtration mode, dye concentration (100 ppm), continues separation for hours and recovery process), higher water flux can be achieved by these ultrahigh MOF loading membrane adsorbers (Fig. 3d).

The membranes reported to date that can realize efficient chiral or protein separation are often based on selective adsorption mechanism rather than size-sieve mechanism. Recovery of chiral compounds or proteins from the washed solvent are needed. Similarly, better performance achieved using these ultrahigh MOF loading membranes adsorbers provides a chance to minimize energy consumption.

Beyond the advantages these new MOF membrane adsorbers had showed, further optimization and study are needed to improve the selectivity and flux especially under complicated water pollution systems (organic, inorganic, biology, etc.). Also, the cost of production and energy consumption should also be further reduced and optimized in a practical water purification system.

In conclusion, a facile and scalable approach, TIPS-HoP, is proposed to fabricate flexible membranes with ultrahigh MOF loading for separation applications. By virtue of their good mechanical behavior, hierarchical porous structure, large surface area and tunable pore chemical environment, ultrahigh flux, and rejection rate for water treatment with the ability to capture small dye molecules were achieved under cross-flow filtration mode. Further tests showed that by altering and/or combining different MOFs, these PE MMM adsorbers can also effectively separate enantiomers and proteins with high selectivity and solvent permeance under dead-end filtration mode. The versatility of TIPS-HoP method makes it easy to fabricate not only PE MMM adsorbers but also zeolite, covalent organic framework (COFs), and other porous solids-based membrane adsorbers with high loadings and diverse functions. It also provides a bright future for large-scale roll-to-roll production of these MOF membrane adsorbers and ease of retrofitting in existing industrial processes.

## Methods

### Material characterization

Powder X-ray diffraction (XRD) analyses were performed on a Rigaku MiniFlex 600 diffractometer with Cu-Kα X-ray radiation (*λ* = 0.154056 nm). Samples were mounted as integral films onto a silicon zero background holder. The PXRD patterns were recorded from 1.5° to 50° (2*θ*) with a step size of 0.02° and scan rate of 10° min^–1^. Fourier-transform infrared attenuated total reflection (FTIR-ATR) spectra were recorded at the range 400–4000 cm^–1^ on a Bruker ALPHA spectrometer. N_2_ adsorption and desorption isotherms were performed at 77 K by using Quantachrome Instrument ASiQMVH002-5. The pore size distributions were calculated using the nonlocalized density functional theory (NLDFT). All samples were tested with nitrogen (99.999%). Field-emission scanning electron microscope (FE-SEM) images were recorded on a FEI Helios NanoLab 600i with 10 kV voltage and 0.2 nA current. The mechanical properties of PE MMMs were investigated with an Instron 5843 system at room temperature. All the PE MMMs were cut into 2 cm × 0.5 cm released along their length at a constant speed of 2 mm min^–1^ by using a 10 N sensor. Streaming potential measurements were made using the SurPASS Electro-kinetic Analyzer (Anton-Paar KG, Graz, Austria) with a flat-plate measuring cell (10 mm × 20 mm). A pH titration was performed at room temperature from pH = 3 to pH = 10 using a 1 mM KCl electrolyte solution. Solution pH was adjusted by the addition of 0.05 M NaOH or 0.1 M HCl at room temperature. The X-ray fluorescence spectrometer (XRF) of NH_2_-UiO-66 PE MMM was quantified on a M4 Tornado system (Bruker Nano, Berlin, Germany) on maps of a 6.5 cm circle, under the following conditions: a Rh tube at 50 kV accelerating voltage and 199 μA beam current with 20 μm distance between spots. The UV–Vis spectrophotometer UV-2600 (Shimadzu, Japan) for molecular absorption quantitative was used to determine the concentration of dyes and proteins (BSA and BHb) before and after filtration. The measurement of chiral optical properties was implemented through the Bio-Logic MOS-450 Circular Dichroism Spectrometer (Bio-Logic, Claix, France) using a quartz cuvette at room temperature. The enantiomeric excess (ee%) value of chiral methyl phenyl sulfoxide was analyzed by high-performance liquid chromatography (HPLC, Shimadzu LC 20 A QA&QC-HPLC-01, Japan) using the CHIRAlCEL OD-H column (4.6 cm × 150 mm (ID × length) × 5 μm, Daicel, Japan). The specific conditions: mobile phase, n-hexane/isopropanol = 90/10 (v/v); flow rate, 1.0 mL min^–1^; detection wavelength, 250 nm; operating temperature, 35 °C. Thermogravimetric (TG) curves were acquired for all samples (~5 mg) in the temperature range 40–800 °C at a heating rate of 10 °C min^−1^ in flowing N_2_ on a Netzsch STA449F5 instrument.

### Synthesis of NH_2_-UiO-66

ZrCl_4_ (pre-dissolved in a DMF/HCl mixture v(DMF):v(HCl) = 5:1) and NH_2_-BDC (pre-dissolved in DMF) at a molar ratio of 1:1.4 were mixed and heated at 80 ^o^C overnight. The obtained powders were isolated by centrifugation and immersed in ethanol for 3 days (the solvent was refreshed every day). Finally, the product was activated under vacuum at 150 ^o^C overnight^36^.

### Synthesis of MIL-100(Cr)

In total, 100 mg of chromium (VI) oxide CrO_3_, 210 mg of H_3_BTC, 0.2 mL of a 5 M HF aqueous solution, and 4.8 mL of deionized water were mixed and stirred for a few minutes. The slurry was then introduced in a Teflon-lined Paar and heated at 493 K for 4 days. The resulting green solid was washed with DI water and acetone and dried at room temperature under air atmosphere. The as-prepared material was degassed under vacuum at 150 ^o^C overnight before further treatment^37^.

### Synthesis of Zn-BLD

A DMF solution (100 mL) containing Zn(NO_3_)_2_·6H_2_O (10 mmol), BDC (5 mmol), and *L*-H_2_lac (5 mmol) in a round bottom flask was heated at 120 ^o^C for 24 h with constantly magnetic stirring. Colorless rod-shaped crystals were collected, washed with DMF and ethanol three times, and then dried at 80 ^o^C to yield Zn-BLD. Finally, the product was activated under vacuum at 90 ^o^C for 3 h^38^.

### Synthesis of BIT-72

AlCl_3_·6H_2_O (930 mg, 3.84 mmol) and 2-hydroxy-1,4-benzendicarboxylic acid (235 mg, 1.29 mmol) were dissolved in 12.5 mL of methanol in a 50 -mL jar. The reaction mixture was heated at 125 °C for 5 h to yield white microcrystalline powder. Then, the solid was washed by deionized water, DMF and CHCl_3_, respectively. Finally, the decanted CHCl_3_ was removed under vacuum to give the product BIT-72^50^.

### Synthesis of MOF-5

Zinc nitrate hexahydrate (4.50 g, 15.0 mmol) and BDC (0.83 g, 5.0 mmol) were dissolved in 490 mL of DMF and 10 mL H_2_O in a 1000 mL jar. The mixture was heated in an oven at 100 °C for 7 h to yield cube-shaped crystals. Then, the crystals were washed with DMF and CHCl_3_, respectively. The decanted and the included CHCl_3_ was removed under vacuum to give colorless MOF-5^51^.

### Synthesis of ZIF-8

2-methylimidazole (3.3 g) dissolved in methanol (70 mL) was mixed with Zn(NO_3_)_2_·6H_2_O (1.5 g) dissolved in methanol (70 mL) and stirred for 24 h. The obtained white powder after filtration was washed repeatedly with methanol and then dried in a vacuum oven at 60 °C. The ZIF-8 was activated at 120 °C under vacuum^52^.

### Synthesis of HKUST-1

H_3_BTC (5.0 g, 24 mmol) and copper(II) nitrate hemi(pentahydrate) (10.0 g, 43 mmol) were stirred for 15 min in 250 mL of solvent consisting of equal parts DMF, ethanol, and deionized water in a 1 L jar. The jar was tightly capped and placed in an 85 °C oven for 20 h to yield HKUST-1 crystals. After decanting the hot mother liquor and rinsing with DMF, the product was immersed in ethanol for 3 days, during which the activation solvent was decanted and freshly replenished three times. The solvent was removed under vacuum at 150 °C^53^.

### Synthesis of Mg-MOF-74

In all, 1.12 g of 2,5-dihydroxyterephthalic acid and 4.75 g of Mg(NO_3_)_2_·6H_2_O were dissolved in 450 mL of DMF, 30 mL of ethanol, and 30 mL of water. The solution was decanted into a 1 L jar, which was capped tightly and placed in a 125 °C oven for 21 h. The products were combined into one batch, and the methanol solvent was decanted and replaced five times over the next 2 days. The sample was evacuated to dryness and activated under vacuum to 150 °C. After 12 h, the samples were cooled to room temperature and stored^54^.

### Synthesis of MOF-808

H_3_BTC (2.1 g, 10 mmol) and ZrOCl_2_·8H_2_O (9.7 g, 30 mmol) were dissolved in DMF/formic acid (450 mL/450 mL) and placed in a 1 L screw-capped glass jar, which was heated to 130 °C for 2 days. A white precipitate was collected by filtration and washed with DMF for 3 days, during which time the DMF was replaced three times per day. Then, the DMF-exchanged compound was filtrated off and immersed in water for 3 days, during which time the water was replaced three times per day. Water exchanged material was then immersed in acetone for 3 days, during which time the acetone was replaced three times per day. Finally, the white powder was activated at 150 °C for 24 h to yield MOF-808^55^.

### Synthesis of MOF-801

Fumaric acid (5.8 g, 50 mmol) and ZrOCl_2_·8H_2_O (16 g, 50 mmol) were dissolved in a solvent mixture of DMF/formic acid (200 mL/70 mL) in a 500 mL screw-capped jar, which was heated at 130 °C for 6 h. The white precipitate was filtrated and washed with fresh DMF and methanol to remove the unreacted metal ions and ligands. Then, the white powder was rinsed three times per day in methanol for 3 days. Finally, the solid was evacuated at 150 °C for 24 h to yield activated sample^56^.

### Preparation of MOF PE MMMs-w%

Take NH_2_-UiO-66 PE MMM-86% as an example, 0.86 g activated NH_2_-UiO-66 were mixed with 0.112 g HDPE and 0.028 g UHMWPE, then, 2 mL of paraffin was added. The mixture was constantly stirred under 200 ^o^C for 20 min to make the powder homogenous. Then the NH_2_-UiO-66 PE MMM-86% were treated with heat and pressure between the two rolls under 120 °C with a speed of 40 rpm. After cooling to the room temperature, the membrane was soaked in 100 mL dichloromethane overnight to wash out the paraffin and dried at room temperature. Membranes with different MOFs or MOF loadings were fabricated using the same method.

### Preparation of MOF MMMs by NIPS

The preparation process of casting solution was as follows: a certain amount of NH_2_-UiO-66 and PAN or PVDF was dissolved in DMAC, the mixture was constantly stirred to be thoroughly homogeneous. Then, the mixture was placed in an oven at 50 °C under vacuum for 2 days to remove the bubbles. After that, the mixed solution was cast on a clean glass plate (30 mm × 20 mm) at room temperature and constant humidity. Finally, the glass plate was immersed in the coagulation bath (water), and then the membrane was peeled from the glass plate and stored in DI water.

### The calculation of permeate flux and rejection

Filtration experiments were performed in a laboratory scale cross-flow filtration system consisting of a filtration cell with an effective membrane area of 3.14 cm^2^ (cross-flow velocity was 70 L h^–1^) (Supplementary Fig. 14a). The total amount of the feed is 2 L and its concentration is 100 ppm and the feed was resupplied by fresh solution (100 ppm) every hour, thus, the feed concentration remained constant during the separation tests. Each tested membrane was initially compacted by the filtration of DI water for 30 min under 0.4 MPa in order to achieve a steady flux. Then, the permeate flux used different dyes as feed solution (100 mg L^–1^) was measured under 0.2 MPa at room temperature and calculated using the following Eq. (1),

1 $${\it{J}} = \frac{{\Delta V}}{{A\Delta t}}$$

where *J* represents the permeate flux (L m^−2^ h^−1^) and *ΔV*, *A*, and *Δt* represent accumulated permeate volume (L), the effective area of composite membrane (m^2^), and filtration time (h), respectively. The dyes rejection of the MMMs-*w*% was defined by Eq. (2),

2 $$R = \left( {{\mathrm{1}} - \frac{{C_{\mathrm{p}}}}{{C_{\mathrm{f}}}}} \right){\mathrm{ \times 100\% }}$$

where *C*_P_ (g L^–1^) and *C*_f_ (g L^–1^) were denoted the concentration of the permeate and the feed solution, respectively. The water-soluble dyes concentration was determined by a UV–Vis spectrophotometer.

### Long-term stability of MOF PE MMM-86%

The long-term stability of PE MMM-86% was evaluated at a specific procedure. First, the Congo red solution (100 mg L^–1^) was filtrated through the membrane for 5 h at 0.2 MPa, and the permeate flux (*J*_P_) was calculated by the weight change over a specific time. Second, the fouled membrane was washed with saturated NaNO_3_ in methanol for 5 min, and the experiment is repeated to test the permeate flux (*J*_R_) of the cleaned membrane. Such cycle was carried out continuously for ten times for each membrane. The flux recovery ratio (FRR) was calculated using the Eq. (3),

3 $${\mathrm{FRR = }}\frac{{J_{\mathrm{R}}}}{{J_{\mathrm{p}}}}{\mathrm{ \times 100\% }}$$

### Adsorption kinetics and capacity of dyes on MOFs

The adsorption kinetics experiments were investigated using CR and CV as model guest solutes. The results were determined by the following method: 5 mg of pristine MOF was mixed with 10 mL dye solution (100 mg L^–1^) in a flask and placed vertically on the table and shaken for a period of time at a speed of 200 rpm min^–1^ to make the solute mixture reach complete adsorption equilibrium.

Batch adsorption experiments were carried out by using a thermostatic shaker at room temperature. For the adsorption, capacity of dyes on MOFs was studied by using the MOF (5 mg) and the dye (50 mL, with a concentration of 10–500 mg L^–1^) and placed in a series of flasks. Then, the flasks were shaken for a 24 h at a speed of 200 rpm min^–1^ to make the solute mixture reach complete adsorption equilibrium. The concentration of dyes was analyzed by a UV–Vis spectrophotometer.

Adsorption kinetics was studied by the second-order equation as nonlinear and linear forms to evaluate the adsorption results of dyes on NH_2_-UiO-66 according to the previously reported (4)^57^.

4 $$\frac{t}{{q_t}} = \frac{1}{{k_1q_{\mathrm{e}}^2}} + \frac{t}{{q_{\mathrm{e}}}}$$

where *k*_2_ (g mg^–1^ min^–1^) is the second-order rate constant. *k*_1_, *R*^2^, and *q*_e_ values of the second-order kinetics were calculated from the plot of *t*/*q*_*t*_ versus *t* and are listed in Supplementary Table 4.

The Langmuir isotherm model can be expressed by the Eq. (5)^57^.

5 $$\frac{{C_{\mathrm{e}}}}{{q_{\mathrm{e}}}} = \frac{1}{{q_{\mathrm{m}}K_{\mathrm{L}}}} + \frac{{C_{\mathrm{e}}}}{{q_{\mathrm{m}}}}$$

where *C*_e_ is the equilibrium concentration of the adsorbate (μmol L^−1^), *q*_e_ is the equilibrium adsorption capacity (μmol g^–1^), *q*_m_ is the maximum adsorption capacity of the adsorbate (μmol g^−1^), and *K*_L_ represents the affinity constant (L μmol^−1^).

### Chiral separation

Chiral separation was performed in a dead-end filtration cell with an effective membrane area of 3.14 cm^2^ (Supplementary Fig. 14b). Acetonitrile contained 0.1 mg mL^–1^ MPS as a feed solution was passed through the MMM at 0.05 MPa. The permeate was collected and evaporated, and the residue was added 1 mL methanol for CD and HPLC analysis. The filtrated MMM was washed with methanol (methanol eluent) for CD spectra measurement, then the membrane was dried under 60 ^o^C in oven for reuse.

The ee% value was calculated from the peak areas of each enantiomer, namely *A*_*R*_ (R-enantiomer) and *A*_*S*_ (S-enantiomer) using the equation below (6),

6 $${\mathrm{ee}}\,{\mathrm{value}}\,\left( \% \right) = \frac{{\left| {A_R\,-\,A_S} \right|}}{{\left| {A_R{{\,+\, A}}_S} \right|}}{\mathrm{ \times 100\% }}$$

### Similar-sized protein separation

MIL-100(Cr) PE MMM-86% membrane was mounted a dead-end filtration cell. First, 3.3 mg of BSA (66 kDa) and 3.3 mg of BHb (65 kDa) were dissolved into the 10 mL of deionized water. Then, 1 mL of mixture was taken from initial solution and re-dispersed into 9 mL of buffer solution at pH = 4.7 (total concentration = 0.066 mg mL^−1^). The separation time was varied from 1 to 5 h with 1 h of the interval. Collected samples at 1–5 h were dried in 70 °C vacuum oven and re-dispersed in the excessive amount of buffer (2 mL).

## Supplementary information

Supplementary Information

Peer Review File

## Source data

Source Data

## Data Availability

The data sets generated during and/or analyzed during the current study are available from the corresponding authors on reasonable request. The data behind Figs. 2–4 is available in the Supplementary Source Data file.
