# Author Correction: Membrane adsorbers with ultrahigh metal-organic framework loading for high flux separations

**DOI:** 10.1038/s41467-025-60965-1

**Published:** 2025-06-23

**Authors:** Hang Wang, Shuang Zhao, Yi Liu, Ruxin Yao, Xiaoqi Wang, Yuhua Cao, Dou Ma, Mingchu Zou, Anyuan Cao, Xiao Feng, Bo Wang

**Affiliations:** 1 Beijing Key Laboratory of Photoelectronic/Electrophotonic Conversion Materials, Key Laboratory of Cluster Science, Ministry of Education, School of Chemistry and Chemical Engineering, Beijing Institute of Technology, Beijing, 100081 P. R. China; 2 PetroChina Research Institute of Petroleum Exploration & Development, Beijing, 100083 P. R. China; 3 Department of Materials Science and Engineering College of Engineering, Peking University, Beijing, 100871 P. R. China; 4 Department of Chemistry, Tsinghua University, Beijing, 100084 P. R. China

Correction to: *Nature Communications* 10.1038/s41467-019-12114-8, published online 16 September 2019

In the version of the article initially published, due to an error in file preparation, the SEM image shown for MOF-5 was a duplicate of the image for Mg-MOF74 in Fig. 2g. A revised Fig. 2 appears below as Fig. 1, and updated Source Data are available alongside this amendment. The original timestamps with date and time of acquisition can be seen at the bottom of each SEM image in the Source Data file. Due to its age, the article cannot be updated directly; this amendment serves to correct the article.

Fig. 1 Corrected Fig. 2
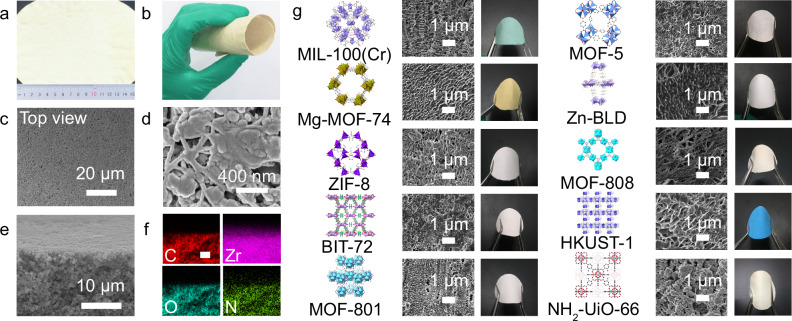


## Supplementary information

Corrected Source Data

