# Supplementary material for: Membrane adsorbers with ultrahigh metal-organic framework loading for high flux separations
Source: Nat Commun. 2019 Sep 16;10:4204. doi: 10.1038/s41467-019-12114-8 (PMC6746862; doi:10.1038/s41467-019-12114-8)
Supplement: Supplementary file 1 — Supplementary Information [file 41467_2019_12114_MOESM1_ESM.pdf]

Supplementary Information

Membrane adsorbers with ultrahigh metal-organic framework loading for high flux separations

Wang et al.

Supplementary Methods

Zirconium tetrachloride ($ZrCl_4$, Energy Chemical Co. Ltd.), zinc nitrate hexahydrate ($Zn(NO_3)_2 \cdot 6H_2O$, Energy Chemical Co. Ltd.), $AlCl_3 \cdot 6H_2O$ (Energy Chemical Co. Ltd.), 2-hydrox-1,4-benzendicarboxylicacid (Energy Chemical Co. Ltd.), ZnO (200 nm, Sigma-Aldrich Co. Ltd.), 200 mesh activated carbon (9 Ding Chemical Co. Ltd), Al_2O_3 (ALADDIN Chemical Co. Ltd.), $CHCl_3$ (Beijing Chemical Reagent Company), copper(II) nitrate hemi(pentahydrate) (Energy Chemical Co. Ltd.), benzene-1,3,5-tricarboxylic acid (H_3BTC, Energy Chemical Co. Ltd.), 2,5-dihydroxyterephthalic acid (Energy Chemical Co. Ltd.), $Mg(NO_3)_2 \cdot 6H_2O$ (Energy Chemical Co. Ltd.), $ZrOCl_2 \cdot 8H_2O$ (Energy Chemical Co. Ltd.), fumaric acid (Energy Chemical Co. Ltd.), formic acid (Energy Chemical Co. Ltd.), 2-aminoterephthalic acid (NH_2-BDC, Energy Chemical Co. Ltd.), 1,4-dicarboxybenzene (BDC, Energy Chemical Co. Ltd.), N,N-dimethylformamide (DMF, Beijing Chemical Reagent Company), trimesic acid (H_3BTC, Energy Chemical Co. Ltd.), ethanol (Beijing Chemical Reagent Company), methyl phenyl sulfoxide (MPS, Energy Chemical Co. Ltd.), polyethylene, high density (HDPE, ALADDIN Chemical Co. Ltd.), polyethylene, ultra-high molecular weight (UHMWPE, Alfa Aesar (China) Chemical Co., Ltd.), paraffin (Alfa Aesar (China) Chemical Co., Ltd.), CrO_3 (Energy Chemical Co. Ltd.), sodium nitrate (Beijing Chemical Reagent Company), L (+)-Lactic acid (Energy Chemical Co. Ltd.), acetonitrile (chromatographic grade, Sigma-Aldrich Co. Ltd.), methanol (chromatographic grade, Sigma-Aldrich Co. Ltd.), bovine serum albumin (BSA, powder form, 66 KDa, Sigma-Aldrich Co. Ltd.), bovine hemoglobin (BHb, powder form, 65 KDa, Sigma-Aldrich), congo red (CR), fuchsine acid (FA), methyl orange (MO), crystal violet (CV), rhodamine B (RB), methylene blue (MB) were purchased from TCI and Sigma Co. Ltd., deionized water (DI water) was produced by Milli-Q water purification system. All chemicals and solvents were used as received without further purification.

Supplementary Figures

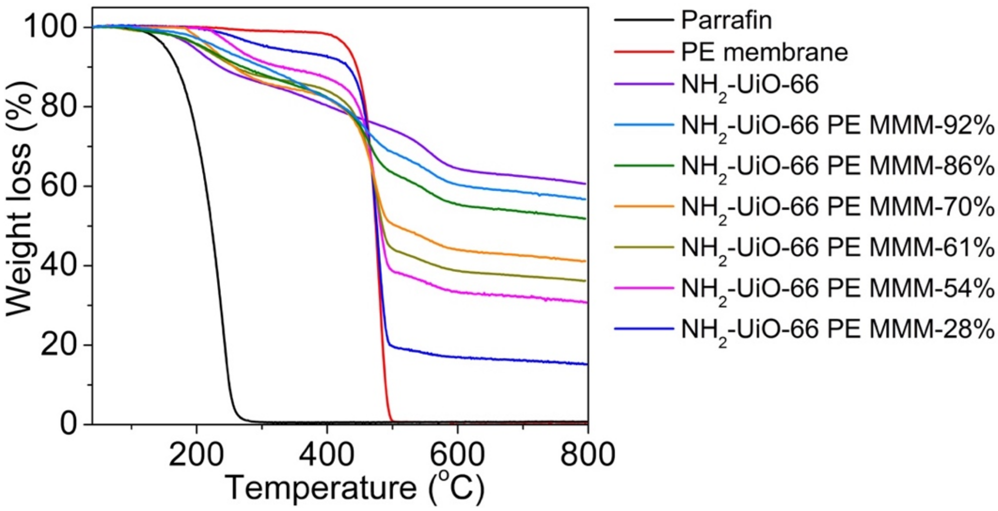

Supplementary Figure 1. TGA curves of NH_2-UiO-66 PE MMMs with different loadings.

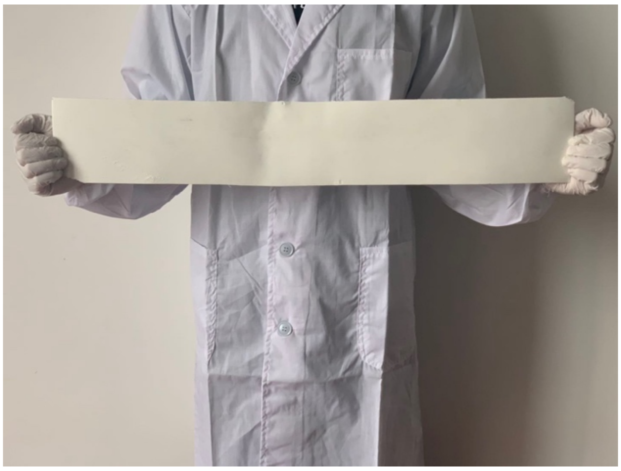

Supplementary Figure 2. Photograph of ZIF-8 PE MMM-86% with an area of 90 cm × 10 cm.

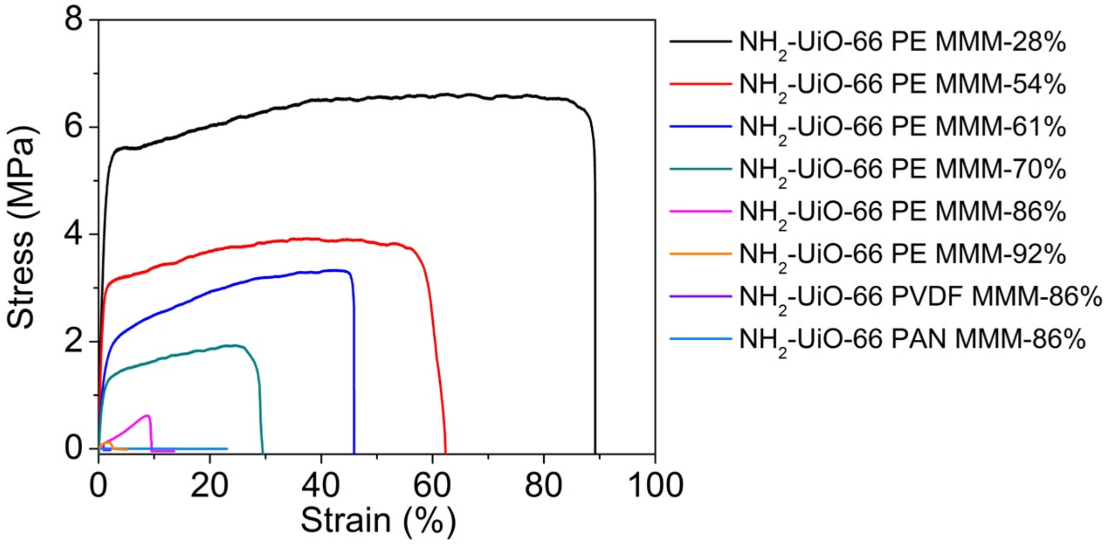

Supplementary Figure 3. The stress-strain comparison of NH_2-UiO-66 PE MMMs with different loadings, NH_2-UiO-66 PVDF MMM-86%, and NH_2-UiO-66 PAN MMM-86%, respectively.

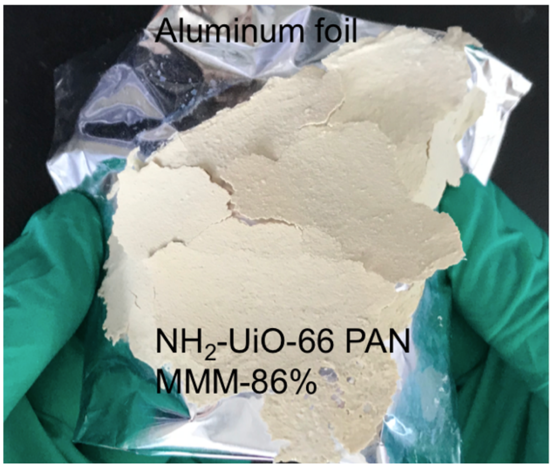

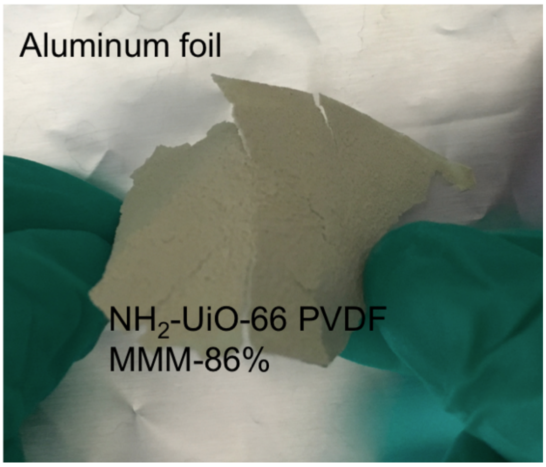

Supplementary Figure 4. Photos of NH_2-UiO-66 PAN MMM-86% and NH_2-UiO-66 PVDF MMM-86% prepared by NIPS method.

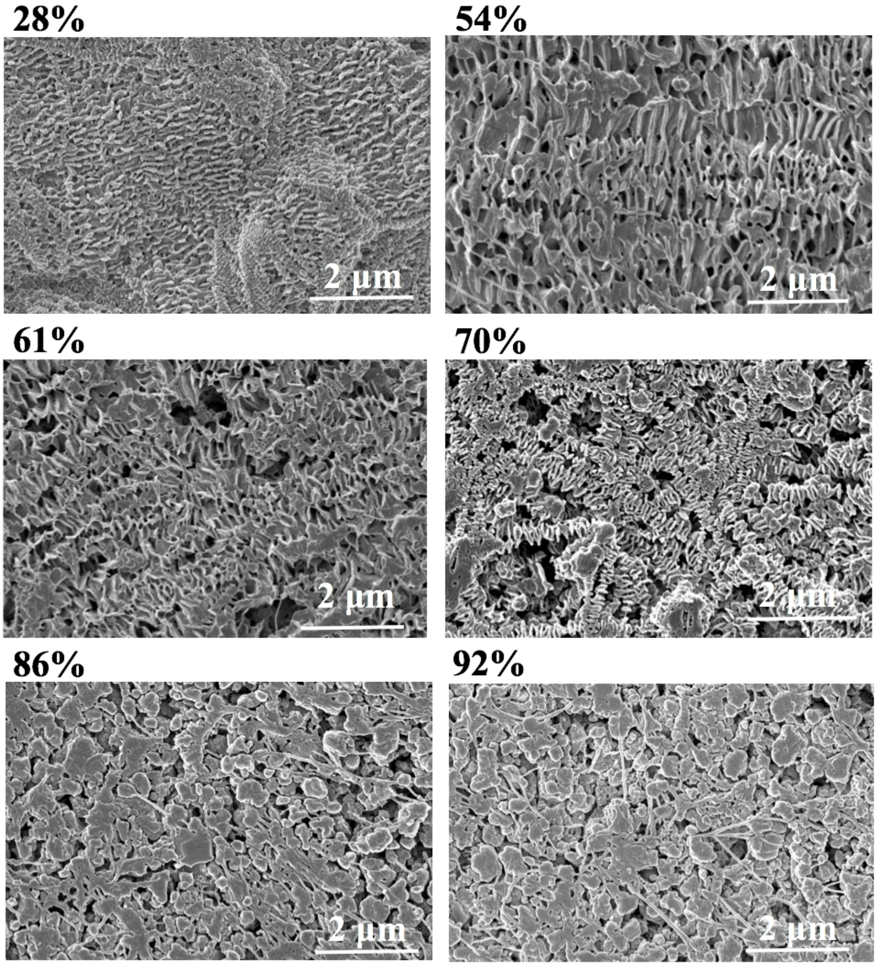

Supplementary Figure 5. Top-view SEM images of the NH_2-UiO-66 PE MMMs with different loadings (scale bar, 2 μm).

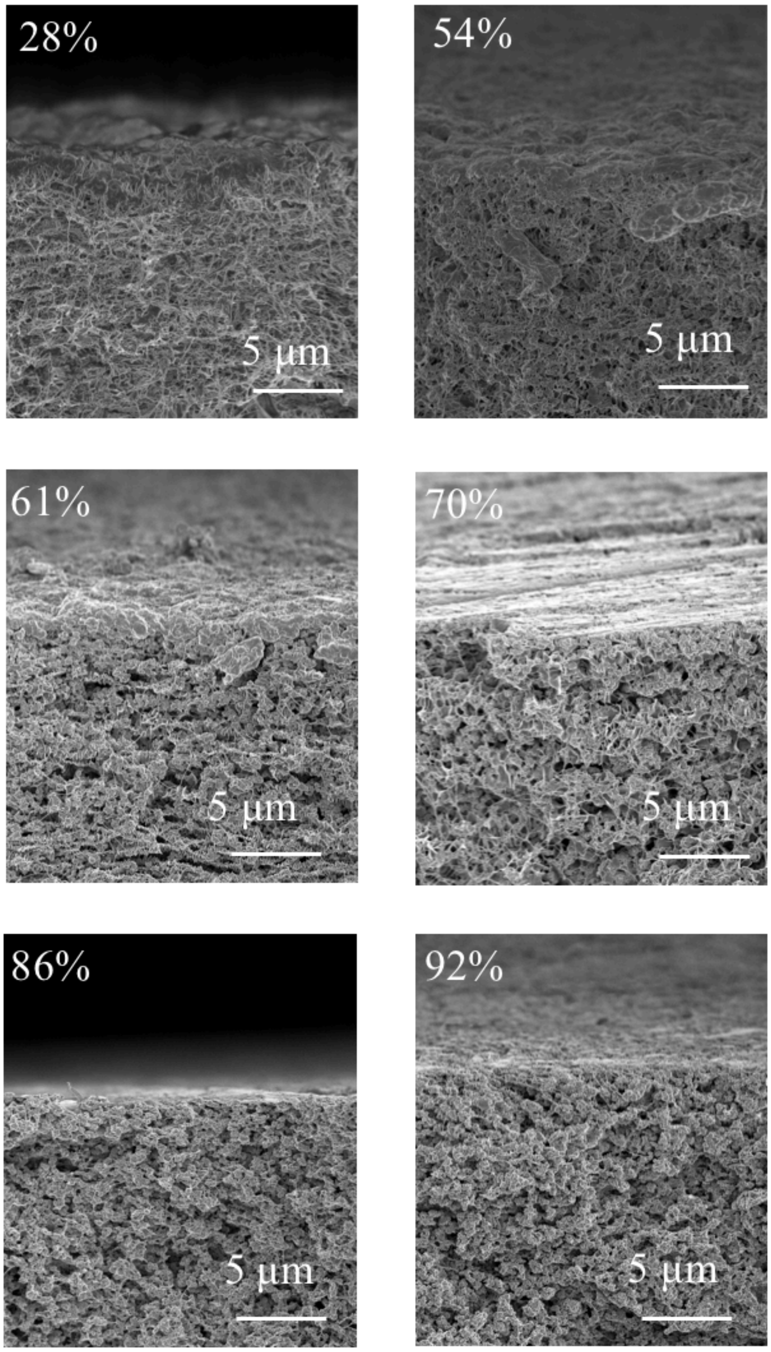

Supplementary Figure 6. Cross-section SEM images of the NH_2-UiO-66 PE MMMs with different loadings (scale bar, 5 μm).

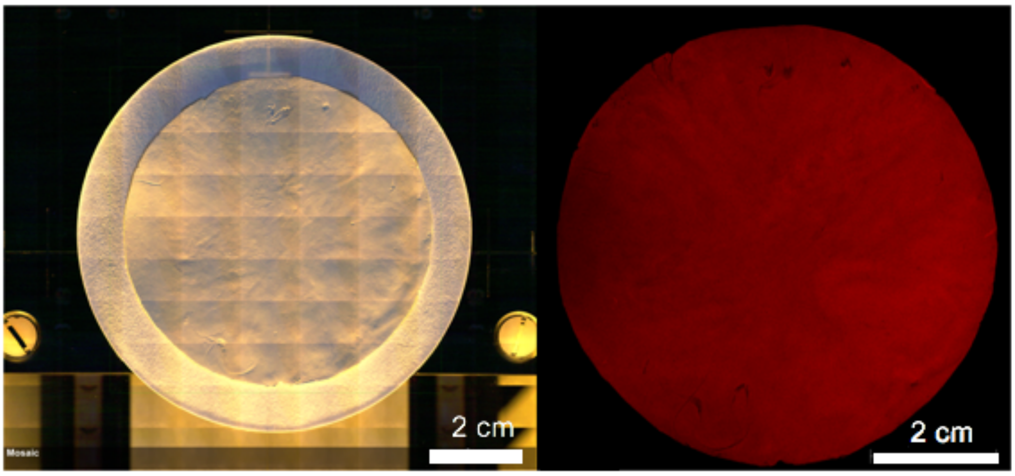

Supplementary Figure 7. Zr mapping for NH_2-UiO-66 PE MMM-86% focused on an area of 28.2 cm^2 (scale bar, 2 cm, diameter: 6.5 cm).

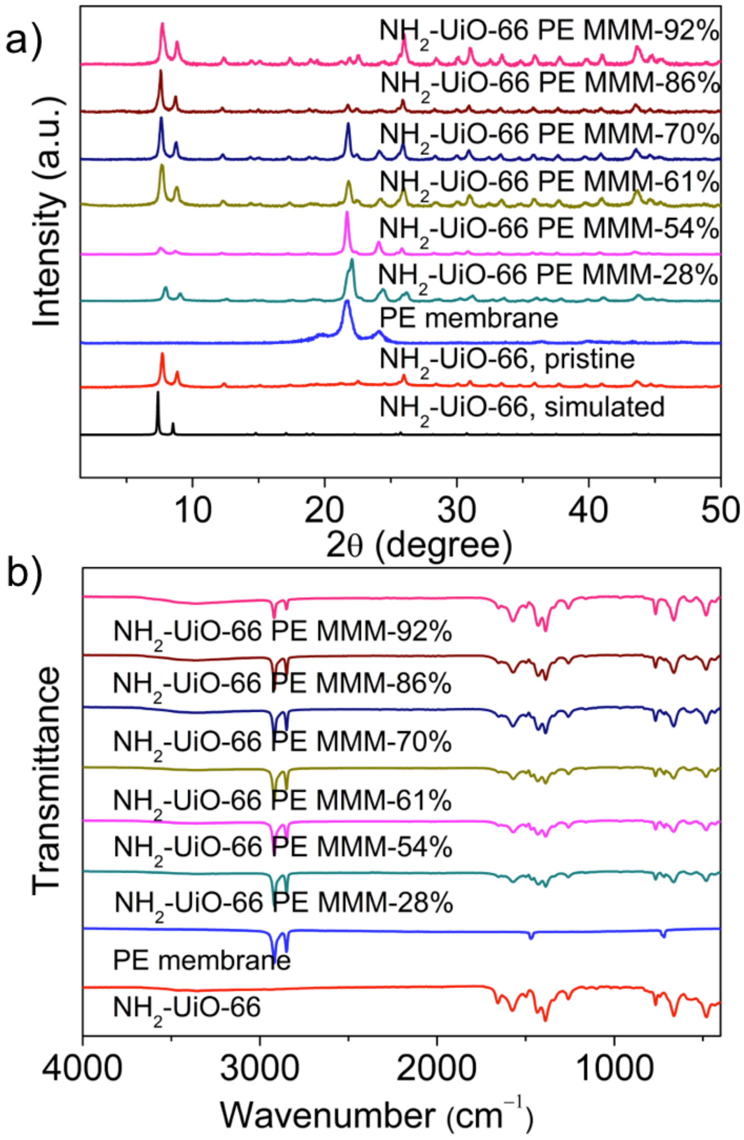

Supplementary Figure 8. PXRD patterns (a) and FTIR-ATR spectra (b) of PE membrane, as-synthesized NH_2-UiO-66, and NH_2-UiO-66 PE MMMs with different loadings, respectively.

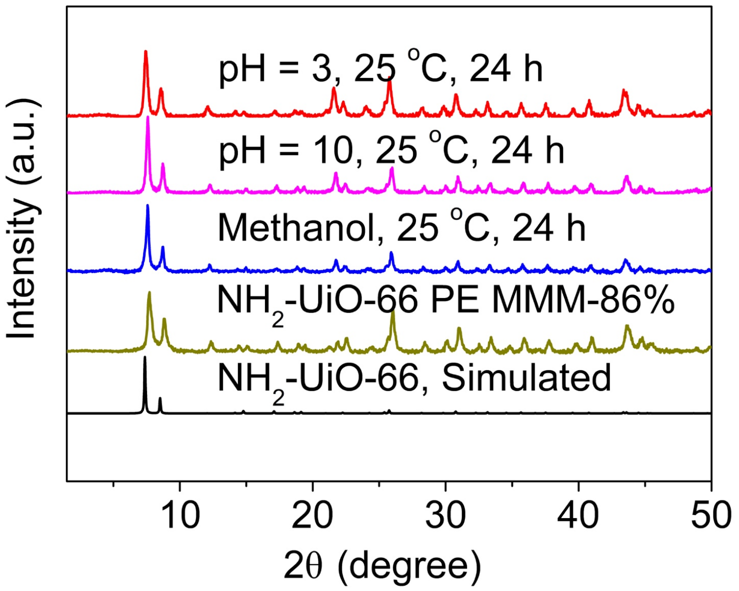

Supplementary Figure 9. XRD patterns of NH_2-UiO-66 PE MMM-86% as-prepared and after stability test.

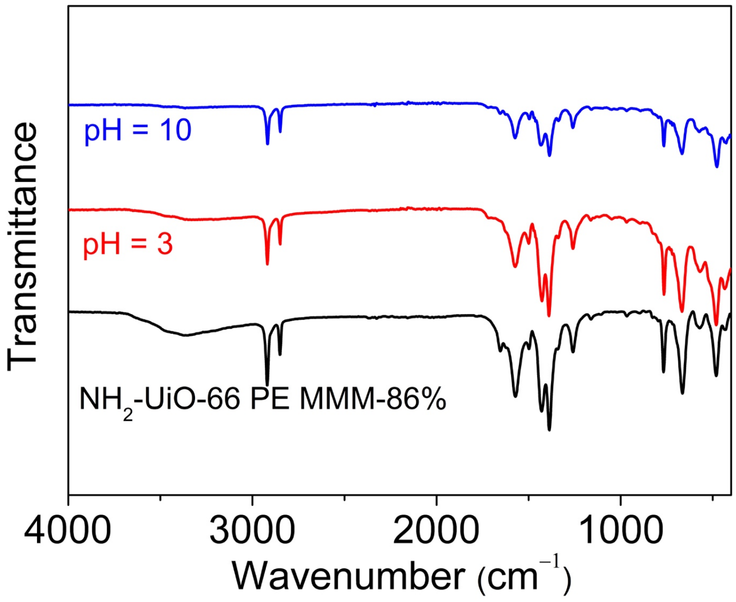

Supplementary Figure 10. FTIR-ATR spectra of NH_2-UiO-66 PE MMM-86% as-prepared and after stability test.

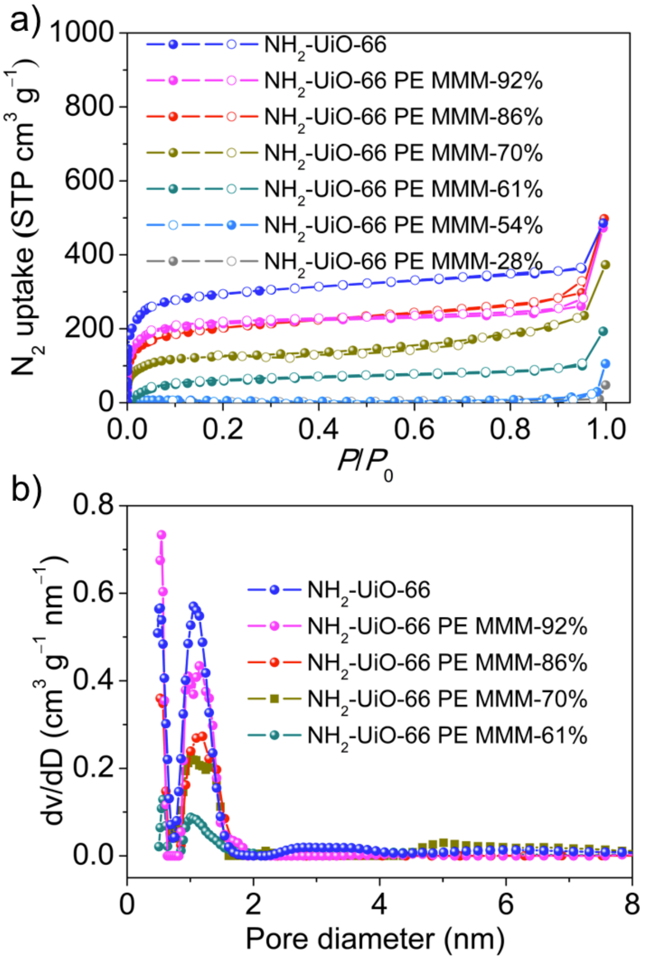

Supplementary Figure 11. N_2 sorption isotherms (a) and pore size distributions (b) of as-synthesized and NH_2-UiO-66 PE MMM with different loadings, respectively.

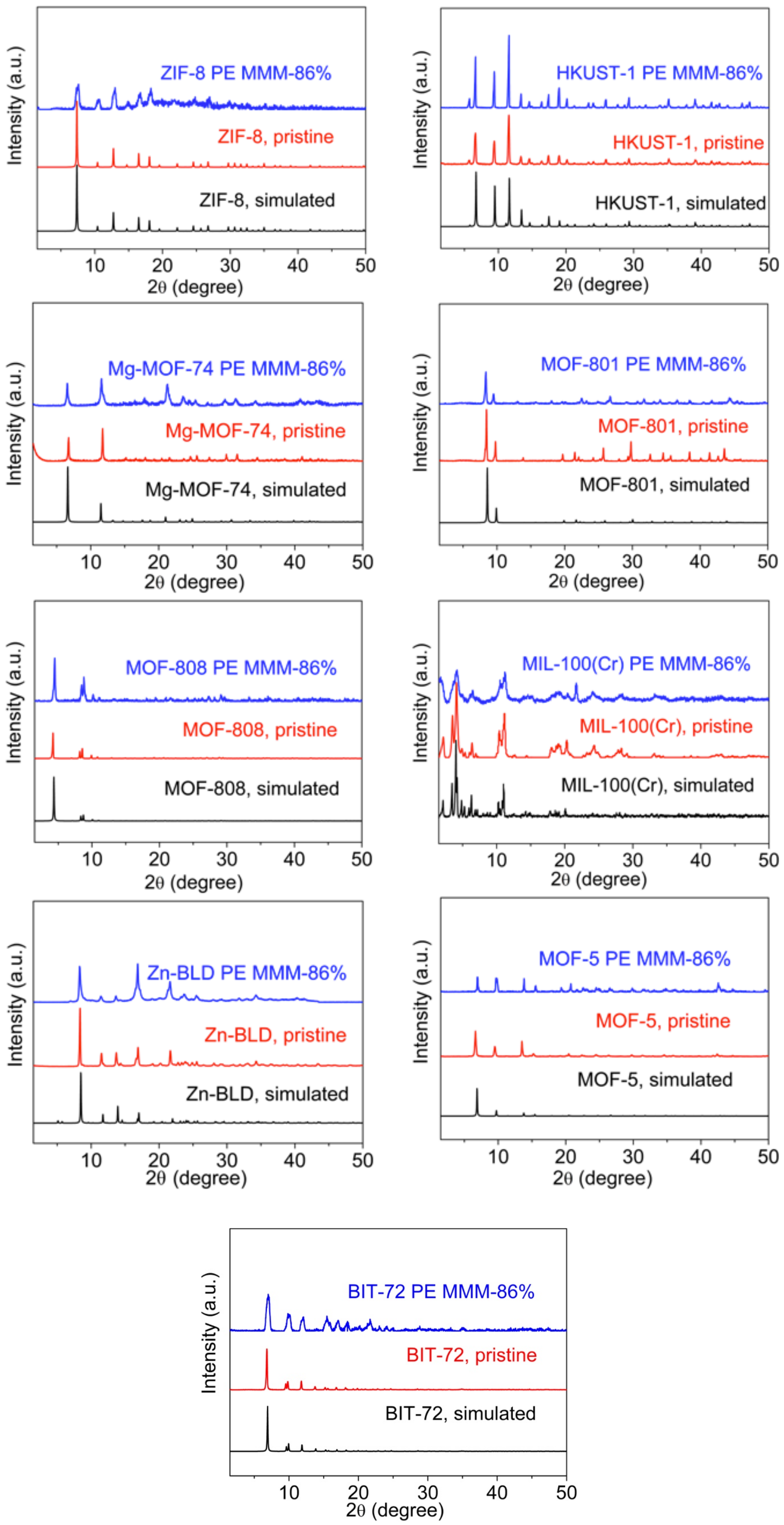

Supplementary Figure 12. PXRD patterns of simulated and as-synthesized ZIF-8, HKUST-1, Mg-MOF-74, MOF-808, MOF-801, MIL-100(Cr), Zn-BLD, MOF-5 and BIT-72 and their corresponding PE MMM with 86% loadings, respectively.

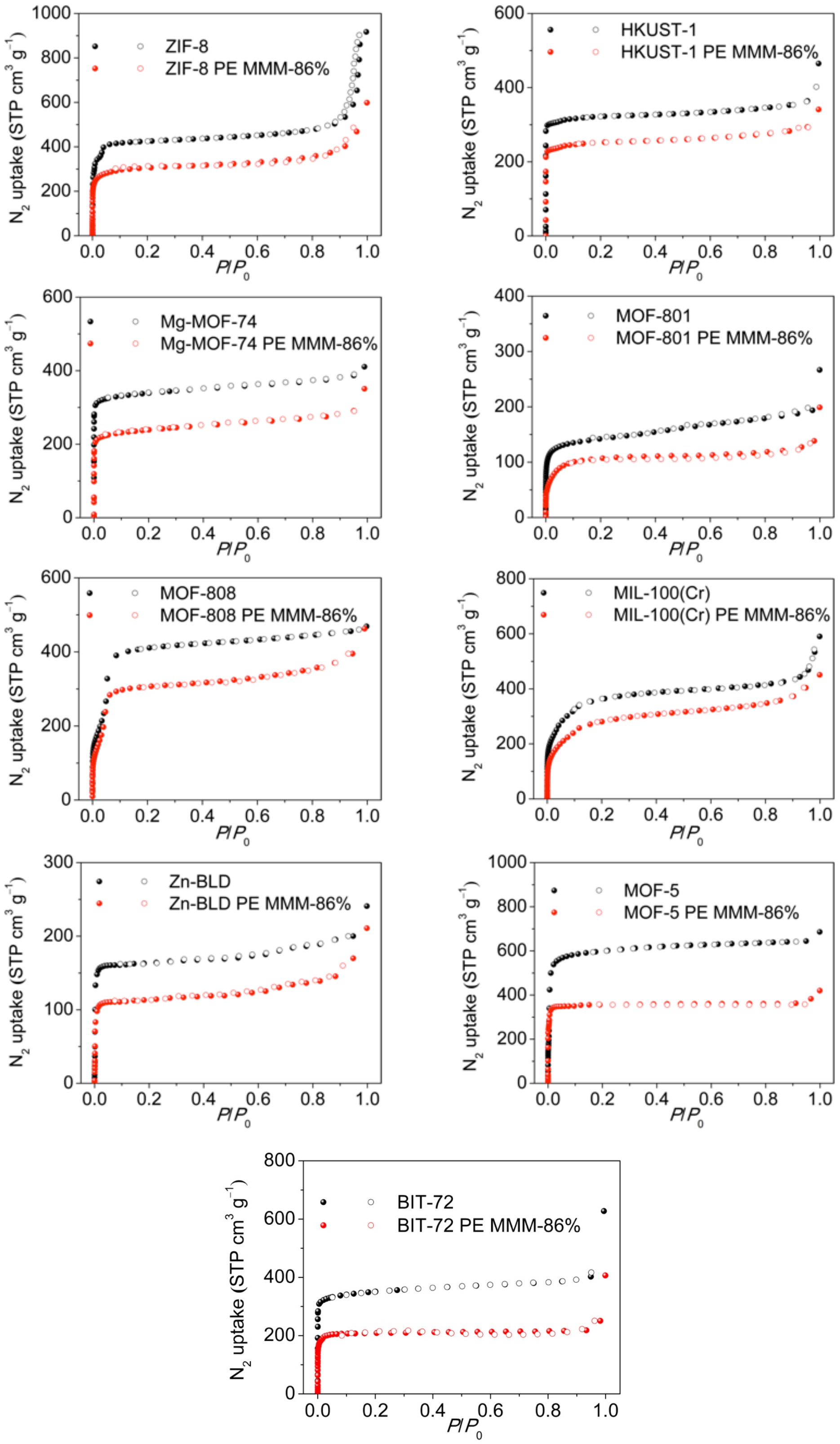

Supplementary Figure 13. N_2 sorption isotherms of as-synthesized ZIF-8, HKUST-1, Mg-MOF-74, MOF-808, MOF-801, MIL-100(Cr), Zn-BLD, MOF-5 and BIT-72, and their corresponding PE MMM with 86% loadings, respectively.

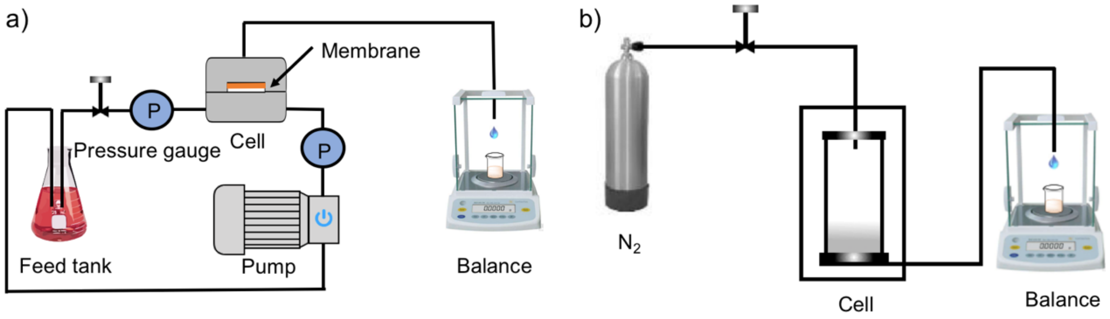

Supplementary Figure 14. Schematic of the apparatus for dyes, racemates and proteins filtration, (a) cross-flow filtration system and (b) dead-end filtration system.

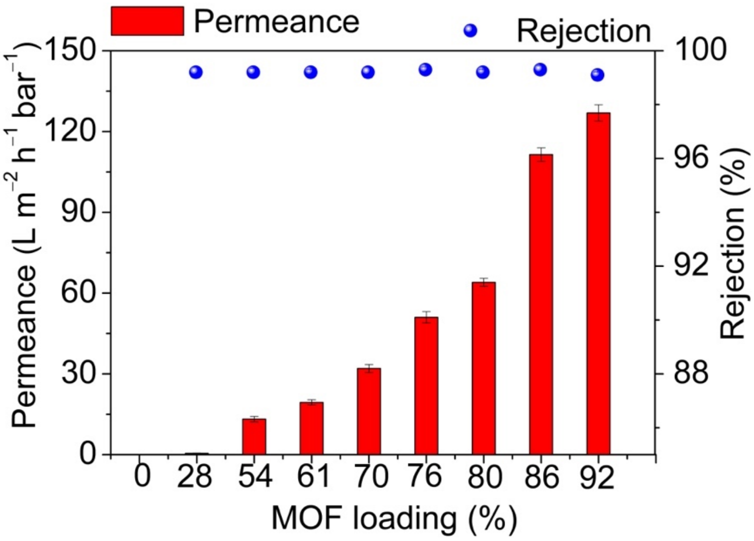

Supplementary Figure 15. The CR permeance and rejection by NH_2-UiO-66 PE MMMs with different loadings, error bars indicate the standard deviation of three independent samples.

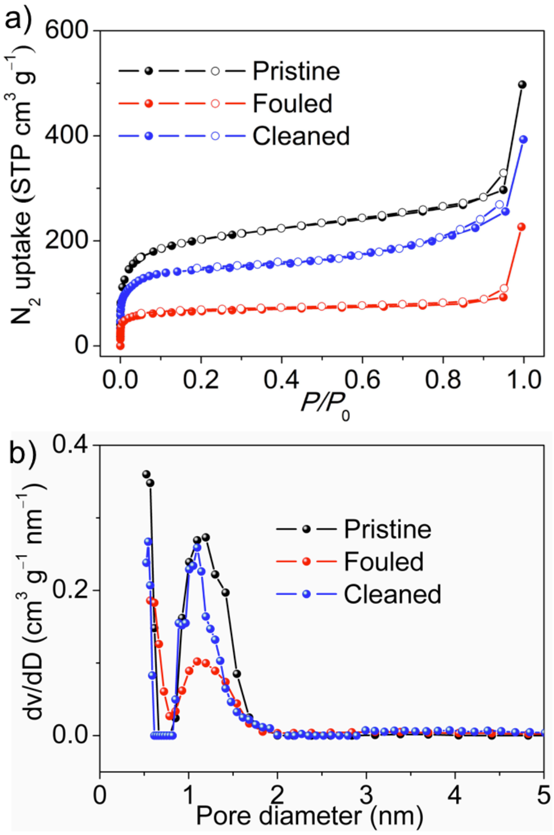

Supplementary Figure 16. N_2 sorption isotherm (a) and pore size distributions (b) of as-synthesized, fouled by CR for 5 h and cleaned NH_2-UiO-66 MOFrane-86%, respectively.

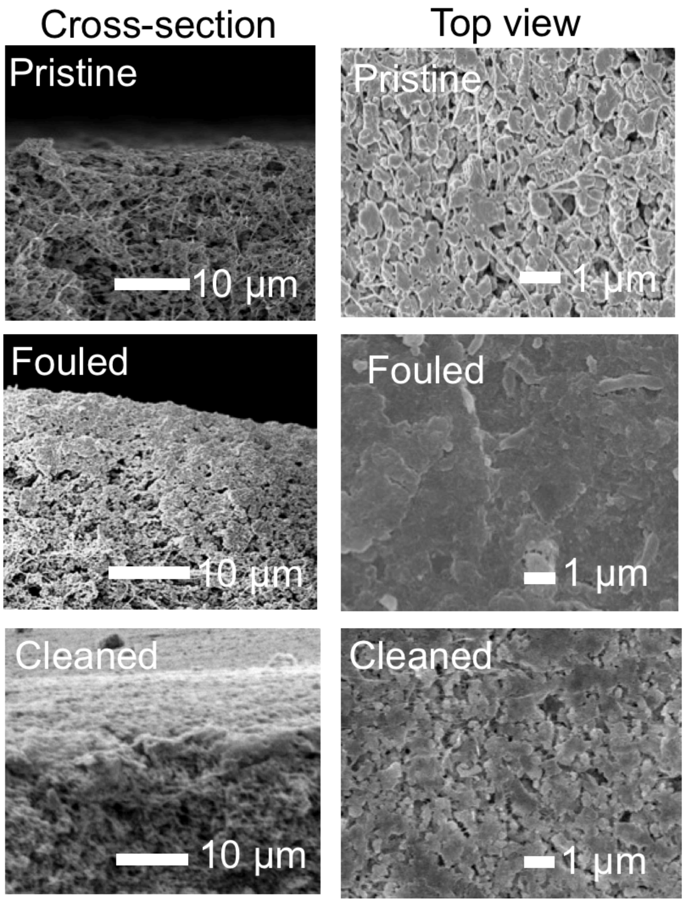

Supplementary Figure 17. Cross-section (left) and top-view (right) SEM images of the pristine, fouled and cleaned NH_2-UiO-66 PE MMM-86% (scale bar, 10 μm for left and 1 μm for right), respectively.

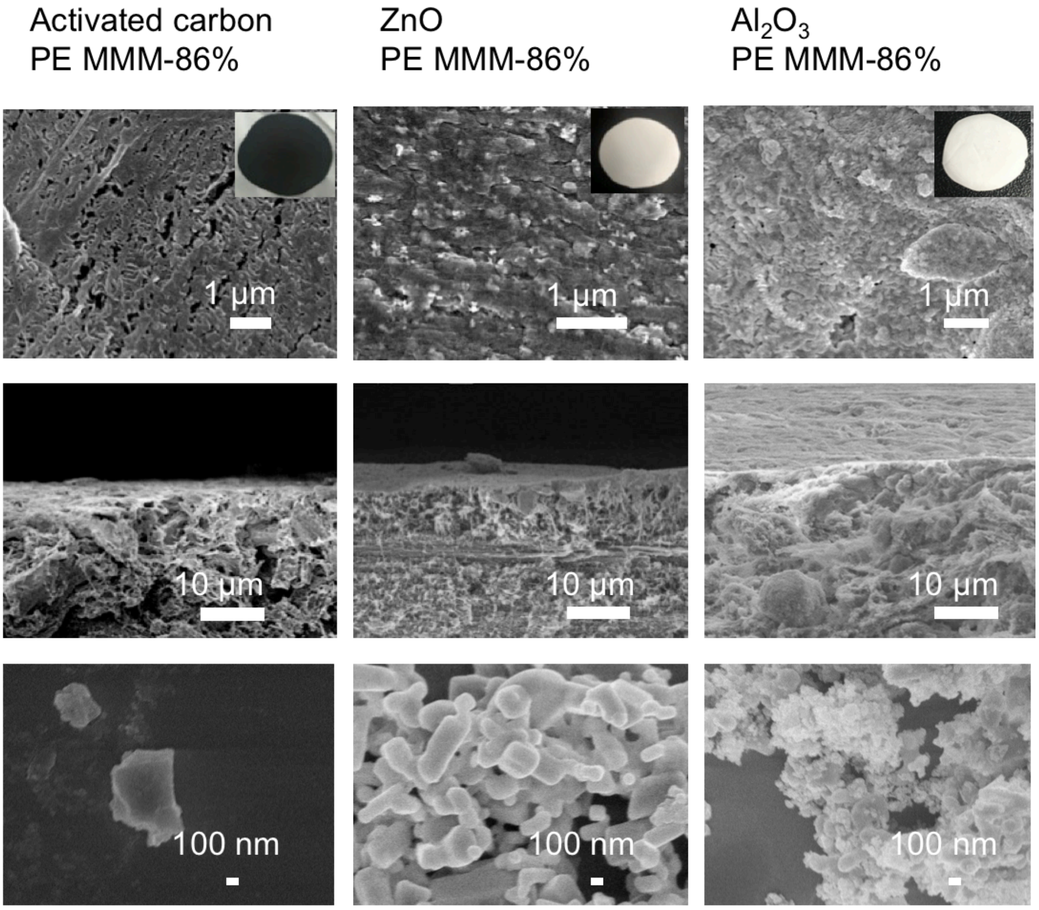

Supplementary Figure 18. Top view SEM images (first line), photographs of the corresponding MMMs (inset), cross-section SEM images (second line) and the combined particles of the corresponding MMMs (third line) of the activated carbon PE MMM-86%, nano-sized ZnO PE MMM-86%, and nano-sized Al_2O_3 PE MMM-86% (scale bar, 1 μm for the first line, 10 μm for the second line and 100 nm for the third line), respectively.

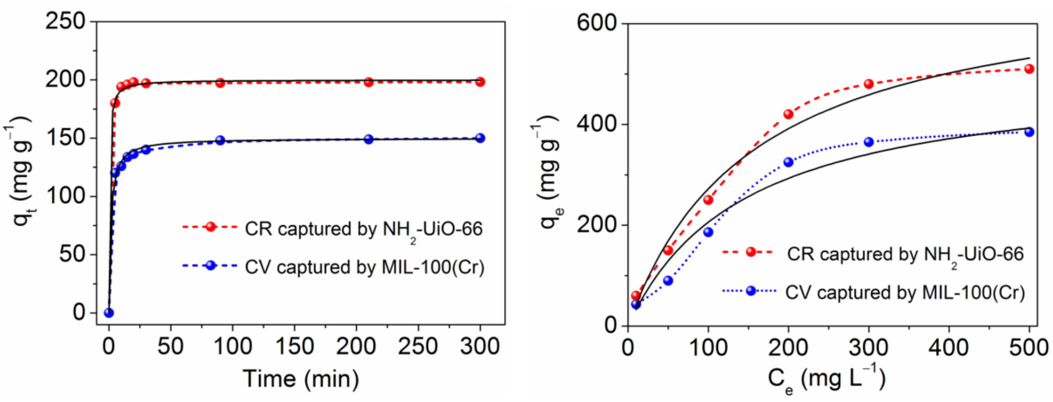

Supplementary Figure 19. Effects of contact time on the adsorption for CR and CV in water by NH_2-UiO-66 and MIL-100(Cr), respectively (left). Adsorption isotherm of CR and CV by NH_2-UiO-66 and MIL-100(Cr), respectively (right).

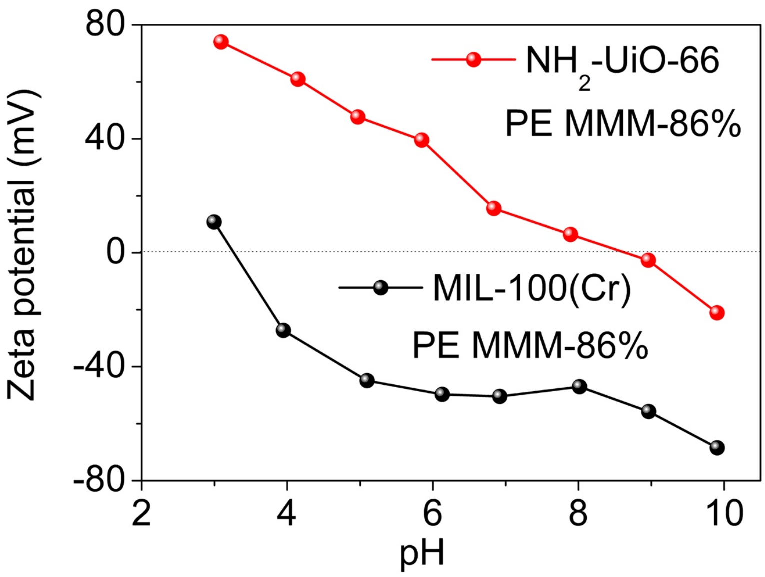

Supplementary Figure 20. Zeta potential of NH_2-UiO-66 PE MMM-86% and MIL-100(Cr) PE MMM-86% at pH values from 3 to 10.

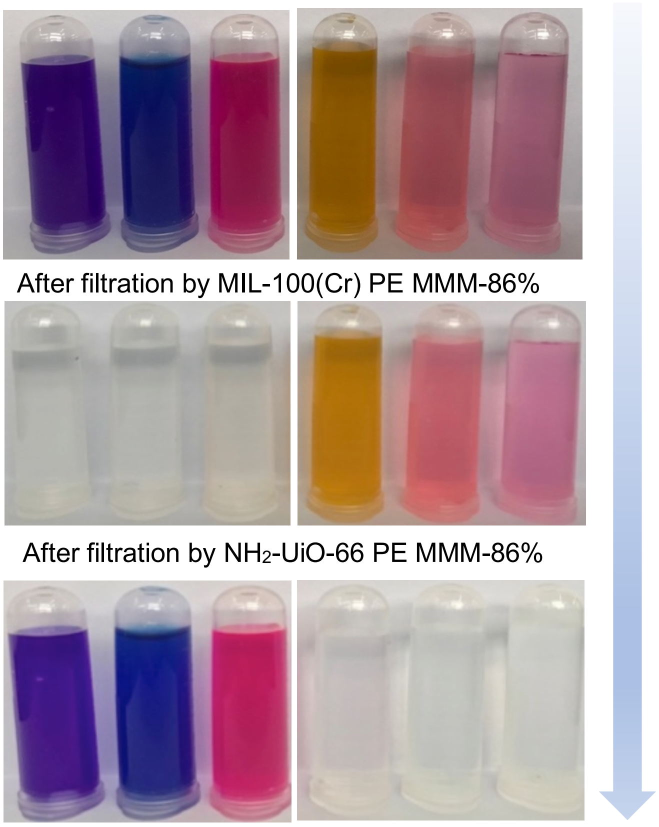

Supplementary Figure 21. Photographs of different dyes feed solutions and permeates filtrated by NH_2-UiO-66 PE MMM-86% and MIL-100(Cr) PE MMM-86%.

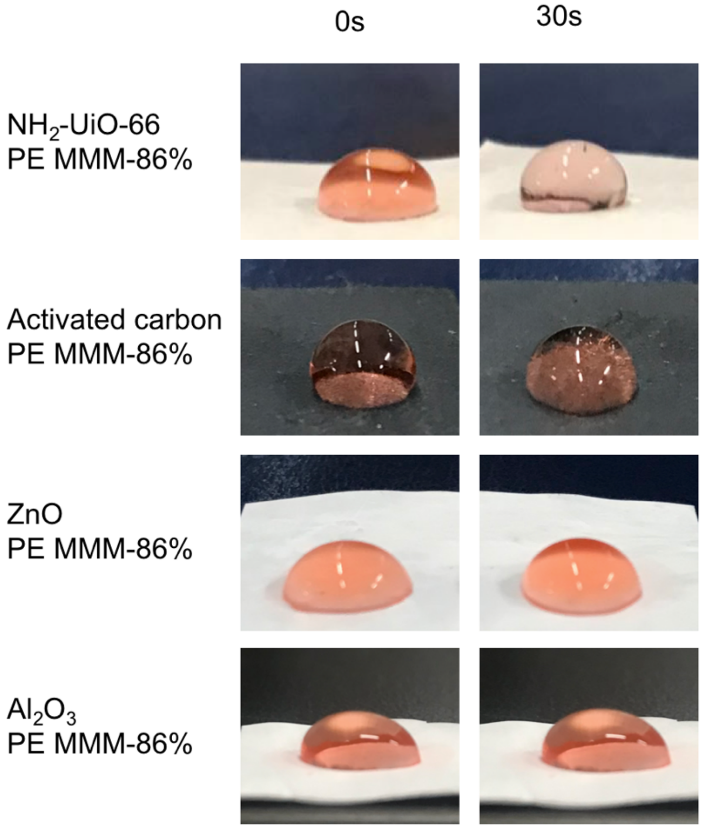

Supplementary Figure 22. Pictures of adding a drop of dye solution onto the surfaces of NH_2-UiO-66 PE MMM-86%, activated carbon PE MMM-86%, ZnO PE MMM-86% and Al_2O_3 PE MMM-86%, respectively.

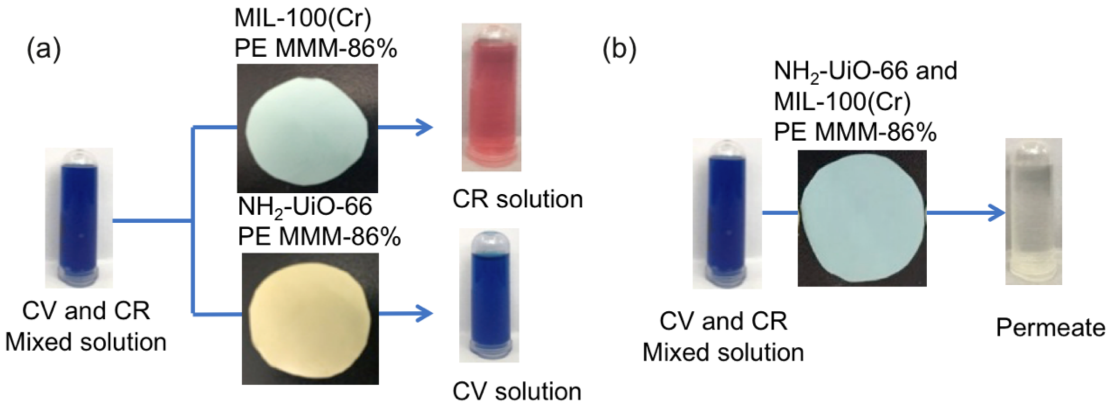

Supplementary Figure 23. (a) The separation of mixed dyes solution using NH_2-UiO-66 PE MMM-86% and MIL-100(Cr) PE MMM-86%. (b) Dyes removal from mixed CR and CV solution by mixed NH_2-UiO-66 and MIL-100(Cr) PE MMM-86%.

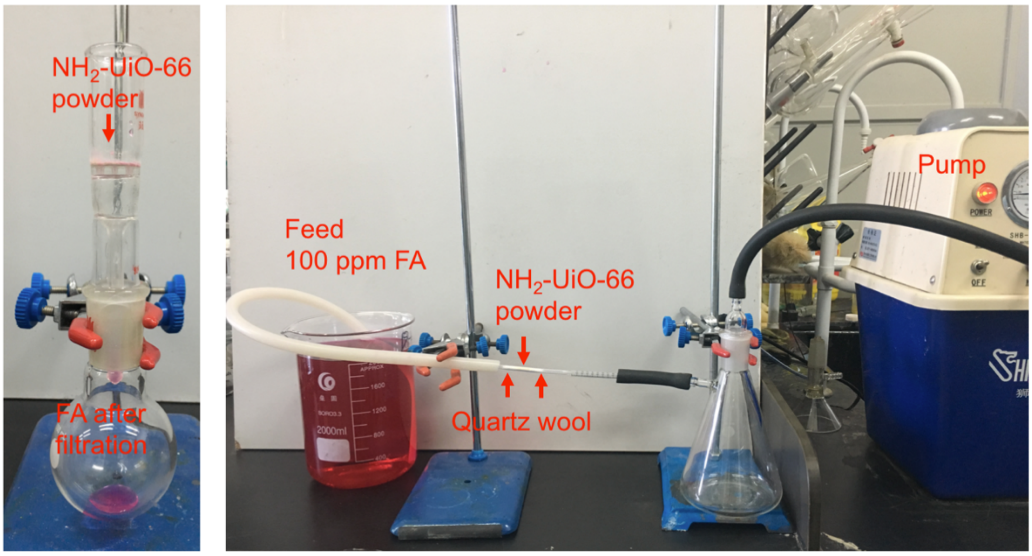

Supplementary Figure 24. The apparatuses for column filtration. NH_2-UiO-66 in the column with the same area (3.14 cm^2) of MMMs used for separation FA (left), NH_2-UiO-66 in the column with a diameter of 6 mm used for separation FA (right).

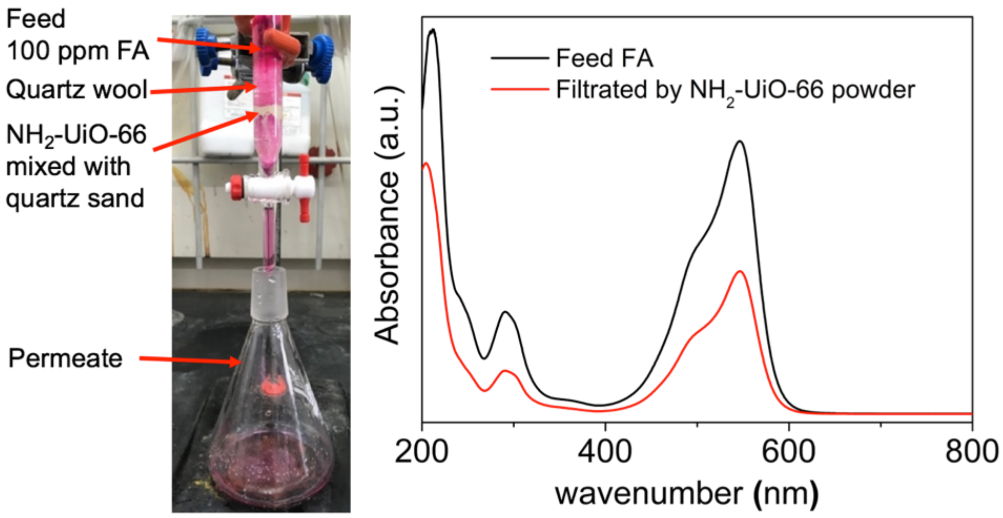

Supplementary Figure 25. The apparatus for dyes filtration by using quartz sand-dispersed MOF particles under atmospheric pressure (left), the UV-vis of 100 ppm feed FA and after column filtration (right).

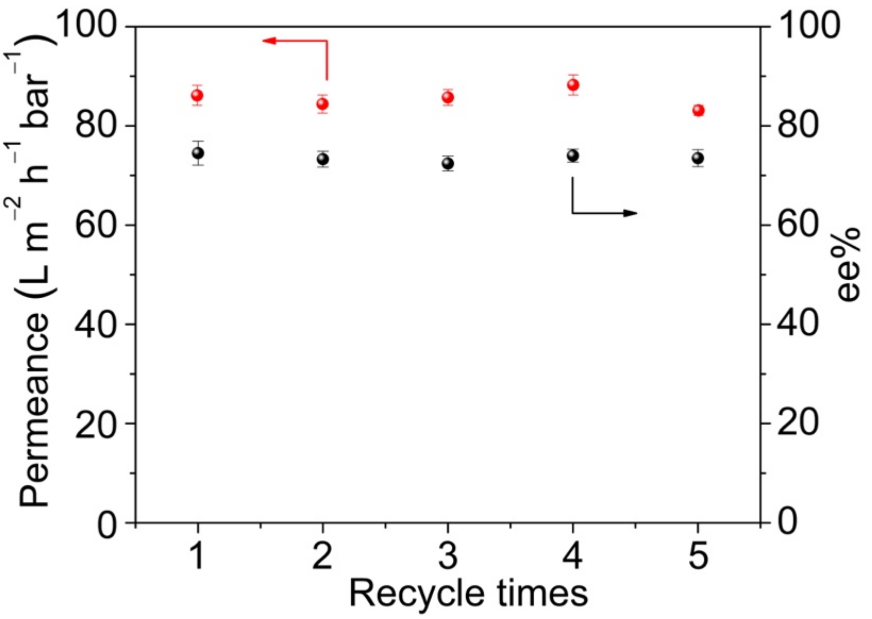

Supplementary Figure 26. The separation performance of Zn-BLD PE MMM-86% for chiral MPS (concentration, 0.1 mg mL^{-1}; applied pressure, 0.05 MPa, error bars indicate the standard deviation of three independent samples).

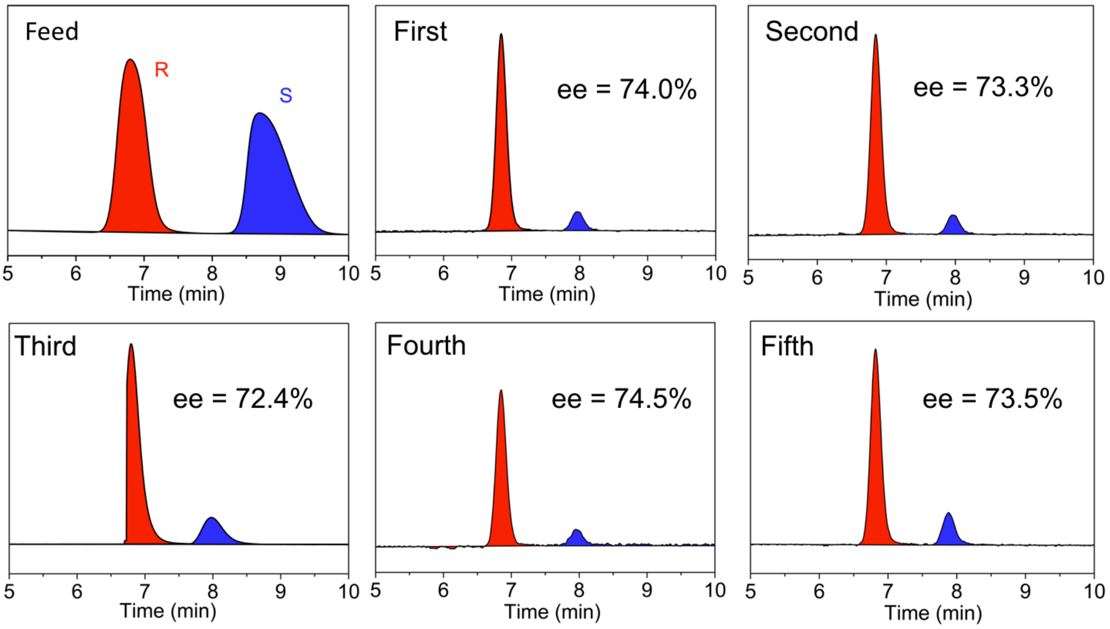

Supplementary Figure 27. High-performance liquid chromatograms of feed chiral MPS and the permeates obtained in five cycles by using NH_2-UiO-66 PE MMM-86% for MPS separation.

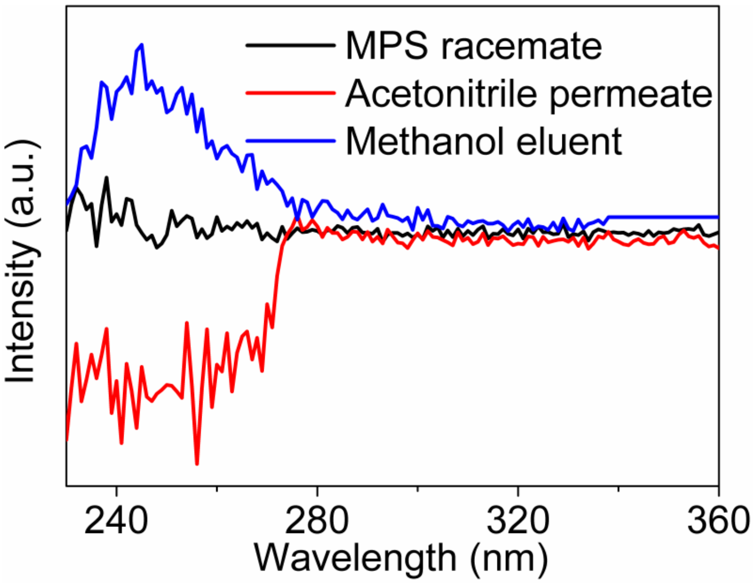

Supplementary Figure 28. Circular dichroism (CD) signals of MPS racemate (black line), acetonitrile permeate (red line) and methanol eluent (blue line). 15 mL acetonitrile contained 0.1 mg mL^{-1} MPS as a feed solution and the filtrated PE MMM was washed with 1 mL methanol.

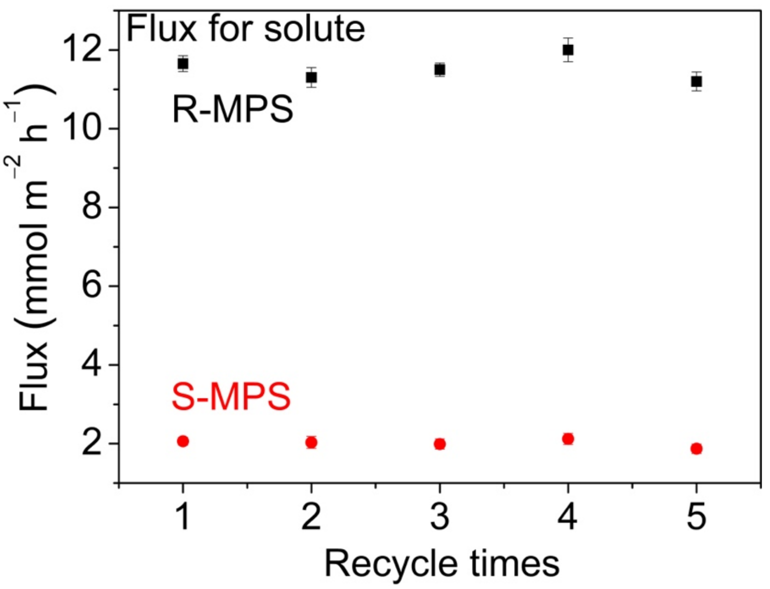

Supplementary Figure 29. The separation performance of Zn-BLD PE MMM-86% for R/S-MPS (concentration, 0.1 mg mL^{-1}; applied pressure, 0.05 MPa, error bars indicate the standard deviation of three independent samples).

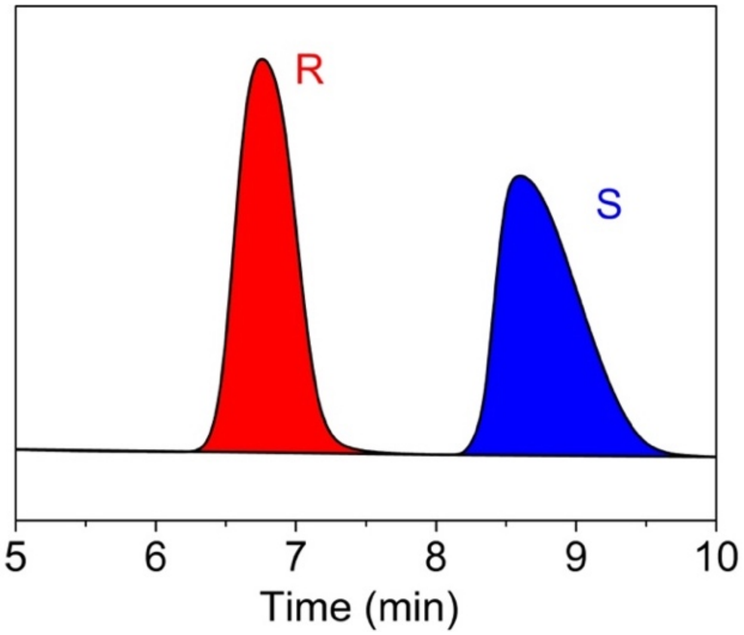

Supplementary Figure 30. High-performance liquid chromatogram of chiral MPS permeate after filtration by NH_2-UiO-66 PE MMM-86%.

Supplementary Tables

Supplementary Table 1. The calculation results of MOF loadings in NH_2-UiO-66 PE MMMs.

MOF loadings (wt%)	Residual mass (%)	Theoretical loadings (%)	Calculated loadings from TGA (%)
0	0	0	0
28	17.6	28	27.6
54	33.1	54	53.3
61	37.5	61	60.4
70	43.1	70	69.4
86	53.5	86	86.1
92	57.1	92	91.9
NH_2-UiO-66	62.1	100	100

Supplementary Table 2. Summary of the strain-stress curve of MOF MMMs.

MOF loadings (wt%)	Strain (%)	Stress (Mpa)
28	79.8	6.5
54	55.4	3.7
61	44.6	3.3
70	25.7	1.9
86	9.0	0.6
92	1.6	0.12
NH_2-UiO-66 PVDF MMM-86%	0.85	0.08
NH_2-UiO-66 PAN MMM-86%	0	0

Supplementary Table 3. Comparison of the theoretical and practical specific surface area of the NH_2-UiO-66 PE MMMs with different MOF loadings.

MOF loading (*w*%)	Calculated surface area based on weight contribution (m^2 g^{-1})	Measured specific surface area (m^2 g^{-1})
0	0	0
28	290	0
54	559	50
61	631	212
70	724	649
86	890	746
92	942	839
Activated NH_2-UiO-66	1035	1035

The differences between the measured specific surface areas and calculated surface area based on weight contributions of individual components indicate that a small number of pores in the MOFs are blocked by the polymers in the PE MMMs, in other words, PE have partially penetrated into pores of MOF particles.

Supplementary Table 4. Summary of the kinetic adsorption of CR and CV by NH_2-UiO-66 and MIL-100(Cr), respectively.

Conditions		Pseudo-second-order rate model		
Adsorbent	Dye	q_e (mg g^{-1})	k_2 (g mg^{-1} min^{-1})	R^2
NH_2-UiO-66	CR	200	0.01	0.998
MIL-100(Cr)	CV	150	0.004	0.996

Supplementary Table 5. Summary of the capacity adsorption of CR and CV by NH_2-UiO-66 and MIL-100(Cr), respectively.

Conditions		Langmuir		
Adsorbent	Dye	q_m (mg g^{-1})	K_L (g mg^{-1} min^{-1})	R^2
NH_2-UiO-66	CR	697.7	0.006	0.983
MIL-100(Cr)	CV	507.7	0.006	0.975

Supplementary Table 6. Summary of the dye adsorption performance by MOF PE MMMs.

MOF PE MMMs-86%	Dyes	Concentration (ppm)	Molecular weight (Da)	Permeance (L m^{-2} h^{-1} bar^{-1})	Rejection
NH_2-UiO-66	OG	100	452.3	115.9 ± 2	99.1 ± 0.1
	CR	100	696.6	102.7 ± 3	99 ± 0.05
	FA	100	585.5	111.4 ± 2.5	99.1 ± 0.08
MIL-100(Cr)	CV	100	407.9	112.5 ± 1.5	99.2 ± 0.15
	RB	100	479	108 ± 1.3	99.3 ± 0.1
	MB	100	319.8	120 ± 1.6	99.0 ± 0.1
Mixed NH_2-UiO-66 with MIL-100(Cr)	CR	100	696.6	116.8 ±1.8	99.1 ± 0.1
	FA	100	585.5	109.8 ± 2.6	98.9 ± 0.15
	CV	100	407.9	117.8 ± 2	99.2 ± 0.08
	RB	100	479	111.2 ± 2.5	98.8 ± 0.08
	MB	100	319.8	125.7 ± 1.5	99. 0 ± 0.1
	OG	100	452.3	103 ± 1.5	99.2 ± 0.05

Supplementary Table 7. The comparison of separation performance.

Membrane	Permeance (L m^{-2} h^{-1} bar^{-1})	Molecular weight cut off (Da)	Rejection (%)	Ref.
NTR-7450	12	Raffinose (504.4)	60%	1
NF-PES-010	10	Raffinose (504.4)	90%	2
NF-270	10.5	Tryptophane (204.2)	99%	2
N30F	11.1	Fasavin CA 73 (742 - 1072)	76.9%	3
Desal-HL-51	9	Raffinose (504.4)	90%	2
Sepro NF 6	12.6	Direct red 80 (1373)	99.9%	4
PEI/LDH	19.8	Methylene blue (319.8)	97.9%	5
ZIF- 8/PSS	25.6	Methyl blue (799.8)	98.6%	6
PEI/GA	25.5	Methyl blue (799.8)	97.3%	7
SG@GO/ ceramic	33	Eriochrome black T (461.3)	98.3%	8
Glycine-LDH	56.6	Eriochrome Black T (461.3)	98.5%	9
BCP membranes	65	Methyl blue (799.8)	99.9%	10
Multi-layered GO	71	Methyl blue (799.8)	99.9%	11
ZIF- 8/PEI	78	Congo red (696.6)	99.2%	12
COF-LZU1	76	Congo red (696.6)	99.2%	13
S-rGO membrane	85.4	Methylene blue (319.8)	98.6%	14
NSC-GO	80	EB (394.3)	85%	15
GO-IPDI	83.3	Methyl blue (799.8)	99.9%	16
TMC-GO	30	Rodamine-WT (566.9)	99.9%	17
Mixed NH_2-UiO-66 and MIL-100(Cr)-PE MMM-86%	117.8	Crystal violet (407.9)	99.2%	This work
	125.7	Methylene Blue (319.8)	99.0%	
	111.2	Rhodamine B (479)	98.8%	
	103	Orange G (452.3)	99.6%	
	116.8	Congo Red (696.6)	99.1%	
	109.8	Fuchsine acid (585.5)	98.9%	
	105	Rose bengal (1017.6)	99.2%	

Supplementary Table 8. Summary of MOF based membranes for enantiomeric separation

MOFs	Substrate	Method	Enantiomeric excess (ee%)	Separation method	Flux for solute (mmol m^{-2} h^{-1})	Flux for solvent (L m^{-2} h^{-1} bar^{-1})	Ref
$Ni_2(L\text{-}asp)_2(bipy)$	Nickel net	Single nickel source	R-MPD/S-MPD (32.5%)	Filtration	N/A	53.3	18
L-His-ZIF-8	AAO	Secondary growth	R/S-1-phenylethanol (76%)	Static diffusion	5.11	N/A	19
$[Zn_2(bdc)(L\text{-}lac)(dmf)]$	Porous ZnO	Secondary growth	R-MPS/S-MPS (33%)	Static diffusion	0.15	N/A	20
$Ni_2(L\text{-}asp)_2(bipy)$	α-Al_2O_3 disks	Secondary growth	R-MPD/S-MPD (35.5%)	Static diffusion	1	N/A	21
$Zn_2(cam)_2(dabco)$	QCM substrate	Layer by layer	R-HDO/S-HDO (21.6%)	Static diffusion	N/A	N/A	23
$[Zn_2(bdc)(L\text{-}lac)(dmf)]$	PE	MMMs	R-MPS/S-MPS (74%)	Filtration	11.5	85	This work

Supplementary Table 9. Summary of membranes for BSA and BHb separation.

Membrane	Selectivity	Ref
PS-b-P4VP	10	24
PCTE/Au/SAMs	67	25
MIL-53(Al)bpy^{+}-d	25	26
(DMAEMA)-g-EVAL	6.2	27
MIL-100(Cr)	94	This work

Supplementary Table 10. The summary of representative works related to the fouling and regeneration process of nanofiltration membranes and other MMMs.

Membrane	Permeate flux (L m^{-2} h^{-1} bar^{-1})	Time interval / Flux attenuation ratio	Flux recovery ratio	Ref
SiO_2-PSS	26.9	2 h / 60%	93%	28
CS-MMT	15.6	2 h / 60%	91%	29
QPEI/PES	12.5	1 h / 40%	94%	30
Fe-phos-(PEI)	11.76	5 h / 30%	70%	31
TiO_2-HMDI	30.5	1 h / 64%	97%	32
PSF/GO	50	3 h / 10%	93%	33
GO/MoS_2	12	0.5 h / 60%	83.4%	34
VES/AgCl–PEI	10	1 h / 35%	74%	35
M-80COOH	30	1 h / 40%	70%	36
PDA/PEI	24	3 h / 50%	90%	37
NH_2-UiO-66 PE MMMs	100	5h / 10%	97%	This work

Supplementary References.

1. Schaep, J., Van der Bruggen, B., Vandecasteele, C. & Wilms, D. Influence of ion size and charge in nanofiltration. *Sep. Purif. Technol.* **14**, 155-162 (1998).
2. Braeken, L. *et al.* Transport mechanisms of dissolved organic compounds in aqueous solution during nanofiltration. *J. Membrane. Sci.* **279**, 311-319 (2006).
3. Cornelis, G., Boussu, K., Van der Bruggen, B., Devreese, I. & Vandecasteele, C. Nanofiltration of nonionic surfactants: Effect of the molecular weight cutoff and contact angle on flux behavior. *Ind. Eng. Chem. Res.* **44**, 7652-7658 (2005).
4. Lin, J. Y. *et al.* Unraveling flux behavior of superhydrophilic loose nanofiltration membranes during textile wastewater treatment. *J. Membrane. Sci.* **493**, 690-702 (2015).
5. Zhao, S. *et al.* Free-standing graphene oxide membrane with tunable channels for efficient water pollution control. *J. Hazard. Mater.* **366**, 659-668 (2018).
6. Zhang, R. *et al.* Coordination-driven in situ self-assembly strategy for the preparation of metal-organic framework hybrid membranes. *Angew. Chem. Int. Ed.* **53**, 9775-9779 (2014).
7. Zhao, S. & Wang, Z. A loose nano-filtration membrane prepared by coating hpan UF membrane with modified PEI for dye reuse and desalination. *J. Membrane. Sci.* **524**, 214-224 (2017).
8. Shen, H. *et al.* Tuning inter-layer spacing of graphene oxide laminates with solvent green to enhance its nanofiltration performance. *J. Membrane. Sci.* **527**, 43-50 (2017).
9. Wang, N. X. *et al.* Tuning molecular sieving channels of layered double hydroxides membrane with direct intercalation of amino acids. *J. Mater. Chem. A.* **6**, 17148-17155 (2018).
10. Shen, Y. X. *et al.* Achieving high permeability and enhanced selectivity for angstrom-scale separations using artificial water channel membranes. *Nat. Commun.* **9**, 2294 (2018).
11. Akbari, A. *et al.* Large-area graphene-based nanofiltration membranes by shear alignment of discotic nematic liquid crystals of graphene oxide. *Nat. Commun.* **7,** 10891 (2016).
12. Yang, L., Wang, Z. & Zhang, J. Highly permeable zeolite imidazolate framework composite membranes fabricated via a chelation-assisted interfacial reaction. *J. Mater. Chem. A.* **5**, 15342-15355 (2017).
13. Fan, H., Gu, J., Meng, H., Knebel, A. & Caro, J. High-flux membranes based on the covalent organic framework COF-LZU1 for selective dye separation by nanofiltration. *Angew. Chem. Int. Ed.* **57**, 4083-4087 (2018).
14. Huang, L. *et al.* Reduced graphene oxide membranes for ultrafast organic solvent nanofiltration. *Adv. Mater.* **28**, 8669-8674 (2016).
15. Huang, H. *et al.* Ultrafast viscous water flow through nanostrand-channelled graphene oxide membranes. *Nat. Commun.* **4**, 2979 (2013).
16. Zhang, P. *et al.* Cross-linking to prepare composite graphene oxide-framework

membranes with high-flux for dyes and heavy metal ions removal. *Chem. Eng. J.* **322**, 657-666 (2017).

17. Hu, M. & Mi, B. Enabling graphene oxide nanosheets as water separation membranes. *Environ. Sci. Technol.* **47**, 3715-3723 (2013).
18. Kang, Z. *et al.* "Single nickel source" in situ fabrication of a stable homochiral mof membrane with chiral resolution properties. *Chem. Commun.* **49**, 10569-10571 (2013).
19. Chan, J. Y. *et al.* Incorporation of homochirality into a zeolitic imidazolate framework membrane for efficient chiral separation. *Angew Chem Int Ed Engl* **57**, 17130-17134 (2018).
20. Wang, W. *et al.* A homochiral metal-organic framework membrane for enantioselective separation. *Chem. Commun.* **48**, 7022-7024 (2012).
21. Huang, K., Dong, X., Ren, R. & Jin, W. Fabrication of homochiral metal-organic framework membrane for enantioseparation of racemic diols. *AIChE J.* **59**, 4364-4372 (2013).
22. Navarro-Sanchez, J. *et al.* Peptide metal-organic frameworks for enantioselective separation of chiral drugs. *J. Am. Chem. Soc.* **139**, 4294-4297 (2017).
23. Liu, B. *et al.* Enantiopure metal-organic framework thin films: Oriented surmof growth and enantioselective adsorption. *Angew. Chem. Int. Ed.* **51**, 807-810 (2012).
24. Qiu, X. *et al.* Selective separation of similarly sized proteins with tunable nanoporous block copolymer membranes. *ACS Nano* **7**, 768-776 (2013).
25. Ku, J. R. & Stroeve, P. Protein diffusion in charged nanotubes: "On-off" behavior of molecular transport. *Langmuir* **20**, 2030-2032 (2004).
26. Jeong, G. Y. *et al.* Metal-organic framework patterns and membranes with heterogeneous pores for flow-assisted switchable separations. *Nat. Commun.* **9**, 3968 (2018).
27. Huang, L. L. *et al.* Similarly sized protein separation of charge-selective ethylene-vinyl alcohol copolymer membrane by grafting dimethylaminoethyl methacrylate. *J. Appl. Polym. Sci.* **135**, 46374 (2018).
28. Xing, L. X., Guo, N. N., Zhang, Y. T., Zhang, H. Q. & Liu, J. D. A negatively charged loose nanofiltration membrane by blending with poly (sodium 4-styrene sulfonate) grafted SiO_2 via SI-ATRP for dye purification. *Sep. Purif. Technol.* **146**, 50-59 (2015).
29. Zhu, J., Tian, M., Zhang, Y., Zhang, H. & Liu, J. Fabrication of a novel “loose” nanofiltration membrane by facile blending with chitosan–montmorillonite nanosheets for dyes purification. *Chem. Eng. J.* **265**, 184-193 (2015).
30. Zhu, J., Zhang, Y., Tian, M. & Liu, J. Fabrication of a mixed matrix membrane with in situ synthesized quaternized polyethylenimine nanoparticles for dye purification and reuse. *ACS Sustain. Chem. Eng.* **3**, 690-701 (2015).
31. Li, P. *et al.* A novel loose-NF membrane based on the phosphorylation and cross-linking of polyethyleneimine layer on porous PAN UF membranes. *J. Membrane. Sci.* **555**, 56-68 (2018).

32. Zhang, L. *et al.* A loose NF membrane by grafting TiO_2-HMDI nanoparticles on PES/β-CD substrate for dye/salt separation. *Sep. Purif. Technol.* **218**, 8-19 (2019).
33. Ji, D. W. *et al.* Preparation of high-flux PSF/GO loose nanofiltration hollow fiber membranes with dense-loose structure for treating textile wastewater. *Chem. Eng. J.* **363**, 33-42 (2019).
34. Zhang, P. *et al.* Novel "loose" GO/MoS_2 composites membranes with enhanced permeability for effective salts and dyes rejection at low pressure. *J. Membrane. Sci.* **574**, 112-123 (2019).
35. Liu, S. *et al.* Chelation–assisted in situ self-assembly route to prepare the loose PAN–based nanocomposite membrane for dye desalination. *J. Membrane. Sci.* **566**, 168-180 (2018).
36. Yuan, S. *et al.* Hydrophilic nanofiltration membranes with reduced humic acid fouling fabricated from copolymers designed by introducing carboxyl groups in the pendant benzene ring. *J. Membrane. Sci.* **563**, 655-663 (2018).
37. Xu, Y., Li, Z., Su, K., Fan, T. & Cao, L. Mussel-inspired modification of PPS membrane to separate and remove the dyes from the wastewater. *Chem. Eng. J.* **341**, 371-382 (2018).
