# Supplementary material for: Membrane adsorbers with ultrahigh metal-organic framework loading for high flux separations
Source: Nat Commun. 2019 Sep 16;10:4204. doi: 10.1038/s41467-019-12114-8 (PMC6746862; doi:10.1038/s41467-019-12114-8)
Supplement: Supplementary file 2 — Peer Review File [file 41467_2019_12114_MOESM2_ESM.docx]

Reviewers' comments:

Reviewer #1 (Remarks to the Author):

This work reports the novel fabrication of MOF/polymer mixed-matrix membranes (MMMs) by melt-processing a variety of MOFs with a mixture of various molecular weight polyethylenes (PE). By using a flow agent, paraffin, the authors produce flowable solutions of MOF and PE that they cast via roll-to-roll processes and cure by thermally-induced phase separation. They then dissolve out the paraffin flow agent using dichloromethane and are left with MMMs at various MOF loadings, up to 92 wt% in polyethylene.

The novelty of this work lies in the technique of melt-processing MOF/polymer MMMs that have been previously inaccessible due to polymer insolubility, and the leap this research makes in moving MOFs into industrial relevance. This point should receive greater emphasis in the manuscript, with less emphasis ascribed to the high flux of the membranes produced. The membrane-forming methods are quite standard, but have not been widely applied in the MOF community. The flux experiments (extensive dye separations, one case of racemate separation, and one of protein separation based on surface charge) are not novel. MOFs have been shown to separate dyes based on electrostatic adsorption in numerous other works, and the MOF used to separate racemates was demonstrated in a previous paper as well. Similarly, the proteins of similar size that were separated, were separated based on electrostatic interactions like the reported dye separation. Through these studies the authors have proved

their MMMs perform like MOFs, in an exciting new accessible form factor. The paper should ideally be restructured to focus on the novel results around the new form factor accessed, and properly contextualize the less-novel separation results. New experiments/applications that specifically address and prove the increased utility of MOFs within MOF/PE MMMs would be ideal to increasing the impact of this work. Finding a new application that only these MMMs can achieve or demonstrating the merit of this fabrication method by comparing with other fabrication methods will improve the novelty of the study.

Specific Comments:

1)	The paper should be copyedited for clarity and understanding; correcting the numerous spelling, grammatical, and word choice errors will greatly increase the clarity of the work. Some examples (not a conclusive list) are given below.

a.	Fix references and punctuation (ref numbers after punctuation).

b.	First sentence of second intro paragraph: word “structs” should be “struts”.

c.	Page 9: “These two membranes exhibit extremely low water flux and almost none CR rejection ability.” – The word “none” should be change to “no”.

d.	Page 9: “OG (99.0% rejection, 1159 L m^–2^ h^–1^ MPa^–1^” is missing a parenthesis at the end.

e.	Page 10 2nd sentence: “removal” should be “remove”.

2)	The lowest loading reported in this manuscript is 28%, however there is no pure polymer membrane synthesized for control purposes. The authors should synthesize a pure polymer membrane and perform the separation experiments.

3)	For the metal oxide controls (Figure S15), are the metal oxides in nanoparticle form? It is hard to determine from the figure. If they are not in nanoparticle form this control is not suitable and would need to be performed with particles on the same size range as the MOFs.

4)	The reviewers propose the addition of thermogravimetric analysis data to assess the actual MOF and polymer content of the MMMs produced. The weight percentages of MOF quoted appear to rely on initial content of MOF, HDPE, and UHMWPE, without including the paraffin. The authors state that the paraffin is dissolved out, yet then report from nitrogen gas sorption that some MOF pores are blocked. If paraffin remains trapped in the MMMs, this would change the overall content of the MMMs produced. If paraffin remains, it should produce a weight loss event distinct from those of the MOF and two polymers, and the actual MOF wt% of the materials (as opposed to the presumed wt%) could be determined, strengthening the results.

5)	The reviewers suggest the authors change the naming scheme of their materials to correspond with literature precedent. These materials are composites of MOF and polymer in membrane form, and the literature standard name for such materials is “mixed-matrix membranes.” The term “mixed-matrix membranes” is used widely in both the MOF field and others that combine ‘filler’ materials with polymer matrices, at a range of filler/matrix ratios. There appears to be no reason for naming these materials differently, and therefore the reviewers suggest they align their naming scheme with standard practice.

6)	Supplementary Figure 1: The depicted stress-strain curves of representative MOF/PE MMMs relative to PVDF and PAN alternatives seems to be incomplete. Much of the following data in the rest of the manuscript compares a range of MOF loadings in PE, yet the lower-MOF content PE MMMs were not assessed for strength. The authors should consider including either comparisons to other literature regarding the strength of their MOF/polymer composites or include a wider range of MOF/PE MMM loadings on this plot to better contextualize the results.

7)	Supplementary Figure 2, 3, 5, 6, 9, 13, 15: Please increase the size of the SEM images provided, in order for readers to properly view the morphological differences referenced in the paper. Please increase the size/resolution of the PXRD traces provided, in order for readers to properly assess fine details in crystallinity changes that are currently not visible.

8)	Please cite previous literature on UiO-66-NH_2_ and MIL-101(Cr)’s ability to separate anionic and cationic dyes. A quick literature search shows that in the below papers, many of the dyes used in this study have been previously studied for adsorption in UiO-66-NH_2_ and MIL-101(Cr). A deeper literature search should be performed by the authors, and any relevant reports should also be included in the references, given the similarity of their conclusions. This section of the manuscript should also be clarified to state that the charge-selective nature of UiO-66-NH2 dye adsorption is well-established. The novelty in this report lies in the fact that it is an MMM performing the separation. For this reason, it would also be enlightening to compare the performance of the MOFs alone vs. the MMMs- is any performance gained within the MMM form that can’t be accessed in the MOF alone? Without emphasis on the utility of the MMM, specifically, there is no novelty in this section of the manuscript.

Yang, Z., Zhu, L. & Chen, L. Selective adsorption and separation of dyes from aqueous solution by core-shell structured nh2-functionalized uio-66 magnetic composites. Journal of Colloid and Interface Science 539, 76-86 (2019).

Yao, B.-J. et al. Post-synthetic polymerization of uio-66-nh2 nanoparticles and polyurethane oligomer toward stand-alone membranes for dye removal and separation. Chemistry – A European Journal 22, 10565-10571 (2016).

Chen, Q. et al. Selective adsorption of cationic dyes by uio-66-nh2. Applied Surface Science 327, 77-85 (2015).

Huang, X.-X. et al. Hierarchically mesostructured mil-101 metal–organic frameworks: Supramolecular template-directed synthesis and accelerated adsorption kinetics for dye removal. CrystEngComm 14, 1613-1617 (2012).

Huang, Y.-F. et al. Facile in situ hydrothermal synthesis of fe3o4@mil-101 composites for removing textile dyes. RSC Advances 4, 47921-47924 (2014).

Wang, T. et al. Facile fabrication of fe3o4/mil-101(cr) for effective removal of acid red 1 and orange g from aqueous solution. Chemical Engineering Journal 295, 403-413 (2016).

9)	We ask the authors to use care in reporting permeance vs. permeability. These are different measurements and appear to be used interchangeably in parts of the document. Specifically, the y-axis of Figure 3d reports “Permeability” values, yet in the text immediately prior to the figure the values are described as such:

“Especially for small molecules with molecular weights close to 300 Da, its water permeance… is almost 3 times higher than that of the reported membranes.”

Please clarify if the reported values are permeability values or permeance values, and comment on the validity of the comparison to reported membranes if these values are not normalized to membrane thickness. Please also include a table of the values with corresponding citations, in the style of Supplementary Table 4, for Figure 3d.

10)	Supplementary Table 4: In the text, the separation of racemates in MOF/PE MMMs is described as excellent in terms of both ee and permeance. Ee values are reported in Supplementary Table 4, but no comparison is given in terms of permeance. Please include those values in the table, elaborate on this point in the text, or exclude this point from the manuscript if it cannot be discussed meaningfully.

Reviewer #2 (Remarks to the Author):

The results claimed in the abstract are impressive with

“flux higher than 1257 L m^–2^ h^–1^ MPa^–1^ and 99 % rejection of small organic dyes (as low as 319 Da)”.

Going through the main text, it is not very clear which is the permeance linked to the rejection of each dye.

To emphasize and make it easier to the readers, please supply in the supplementary information a table listing permeance (L m–2 h–1 bar–1) clearly linked to the rejection of each dye used for each MOF/polymer combination. Please add the error bars for these measurements.

Please note that “permeance” is the most appropriate term. Use preferentially unities with bar, which are the most used in the field and would be easier to directly compare.

Many groups are working on mixed matrix membranes, integrating MOFs in a polymeric matrix. A big challenge is the compatibility between the MOF and the polymer. A poor interaction might lead to cavities at the MOF-polymer interface and to an apparent high permeation. A second frequent issue when testing the membranes with dyes is a high adsorption on the membrane surface, leading to an apparent high rejection.

To avoid having adsorption as cause of an apparent rejection, please report also the concentration values of the feed, retentate and permeate for the performance measurements.

UHMWPE gives a good mechanical property but the extrusion might be challenging, the melt viscosity is high and the interaction with the MOFs could be poor.

Please provide microscopy images at higher magnification, detailing the interface between the MOF crystals and the polymer.

Reviewer #3 (Remarks to the Author):

This paper reports the manufacturing of MOF-based membranes with hierarchically porous structures and demonstrated the applications for dye removal and separation of chiral molecules and proteins. The authors claimed that their MOFanes are Mixed Matrix Membranes (MMMs) and compared their results with other nanofiltration and molecular sieving membranes (reference 25, 29-47). However their claims appear somewhat misleading and really compromise the quality of the work.

The membranes reported in this work are membrane adsorbers or membrane sorbents. In the membrane industry, membrane-based adsorbers or sorbents have been developed for a wide range of separation applications. Similarly, membrane chromatography methods have been developed for chemical separation and protein purification. Membrane adsorbers combine the advantages of adsorption and membrane separation, giving high flow rates and low pressure drop. Therefore, it is not surprising that this paper obtained high water permeance or solvent permeance. Throughout the paper the authors did not cite any literature on membrane adsorbers or membrane chromatography. They did not introduce the relevant progress of this field, and the key challenges to be solved in this study.

The working principles of membrane absorbents are different from those of mixed matrix membranes. In mixed matrix membranes, the MOF-based fillers are expected to facilitate the transport of molecules (although the mechanism can be complex). In membrane adsorbers with high loading of adsorbents such as MOFs, the adsorption of species on surfaces and pores of MOFs becomes dominant, and regeneration of the chemicals adsorbed in the sorbents. These features differentiate the membranes from conventional RO and nanofiltration membranes. Therefore, the authors cannot simply compare the flux and selectivity and claim the superior performance with other separation membranes.

The application of membrane adsorber for removal of dye molecules is not a new idea. Recent studies have shown that MOF membrane adsorbers can efficiently adsorb and remove dye molecules and give high water permeance of 20,000 L m-2 bar-1 h-1, which is much higher than the water permeance achieved in this study (120 L m-2 bar-1 h-1). MOF nanocrystals can be deposited in porous alumina supports or polymer substrates such as PTFE. The authors need to review the previous works and re-evaluate the novelty of this work.

References:

Membrane adsorber containing new Sm(III)–organic framework for dye removal. Environ. Sci.: Nano, 2019, Doi:10.1039/C9EN00018F.

High-permeance metal–organic framework-based membrane adsorber for the removal of dye molecules in aqueous phase. Environ. Sci.: Nano, 2017,4, 2205-2214.

Metal–organic frameworks for dye sorption: structure–property relationships and scalable deposition of the membrane adsorber. CrystEngComm, 2018,20, 5465-5474.

Certainly, there are some interesting results reported in this work. The authors report a new method for manufacturing membrane adsorber using high molecular weight polymer binder and high loading of MOFs with accessible porosity. The authors evaluated a series of MOFs and dye molecules with different size and charges, and showed that the charge effect plays a significant role in adsorption. However, the results provided in the paper are insufficient to support their claims. It is not clear whether the molecules are adsorbed on the surface or within the micropores and how the pore structure of MOFs determine the separation performance.

The authors need to compare the separation efficiency with conventional adsorbents. For example, if the same polymer supported MOF particles are packed into one column and the dye solutions are purged through the column, what is the separation performance?

How will the membrane reduce energy consumption compared to adsorption technology.

Rejection is not a proper parameter to characterise the purification of dye molecules in membrane adsorber because it strongly depends on the feed concentration. The adsorption capacity of dye molecules in MOFs is more important. The authors used a variety of MOF materials but there is not a clear connection between the MOF structures and the adsorption performance. The dye molecules may be adsorbed on the surface of the MOFs. To examine this effect, the authors can compare the MOF nanocrystals and large micron-sized crystals.

For the chiral and protein separations in the dead end cells, were these adsorption or dynamic separation? The chiral separation data are not supported by evidence. Whether the membrane adsorber is intrinsically chiral and how the adsorption of chiral molecules can be used to separate the chiral molecules. The authors only provided one enantiomeric excess value and solvent flux and one HPLC curve. What was the enantiomer permeance or flux? What about the other membranes without chirality? What about the value of ee as a function of time?

The protein separation results are also questionable. Only one selective factor was derived from the protein concentration profiles. The authors attributed to the separation to electrostatic attraction without fundamental understandings.

The authors claimed that “and this roll-to-roll production can be easily scaled up by retrofitting in current industrial facilities and processes”. No evidence was provided – diagram shows roll to roll, but it is not clear whether the authors really performed this in reality. The size of 15cm2 x 13 cm2 is quite small for roll-to-roll manufacturing. From the supplementary information it seems that only single sheets were made. A detailed description of the equipment used for the roll to roll process, the sizes of membranes produced, is required. The authors did not mention the thickness of the membranes produced.

Diagrams of equipment in Supplementary Fig 11 are inadequate – no dimensions or volumes provided. How is pressure maintained in the crossflow cell? No backpressure valve reported.

English needs to be improved – no such word as “structs”. Consumption should be ‘assumption’, ‘able to removal’ to ‘able to remove’.

**Responses to referees' comments
Referee 1:**

1. *This work reports the novel fabrication of MOF/polymer mixed-matrix membranes (MMMs)…The novelty of this work lies in the technique of melt-processing MOF/polymer MMMs that have been previously inaccessible due to polymer insolubility, and the leap this research makes in moving MOFs into industrial relevance. This point should receive greater emphasis in the manuscript, with less emphasis ascribed to the high flux of the membranes produced. Through these studies the authors have proved their MMMs perform like MOFs, in an exciting new accessible form factor. The paper should ideally be restructured to focus on the novel results around the new form factor accessed, and properly contextualize the less-novel separation results. New experiments/applications that specifically address and prove the increased utility of MOFs within MOF/PE MMMs would be ideal to increasing the impact of this work. Finding a new application that only these MMMs can achieve … comparing with other fabrication methods will improve the novelty of the study.*

Per suggestion, we have reconstructed the manuscript by emphasizing the novel methodology and demonstrating by comparing the results and merits. Specifically, we have thoroughly investigated the characteristics and advantages of our thermally induced phase separation-hot pressing (TIPS-HoP) technique in comparison with all the reported relevant fabrication methods, including nonsolvent induced phase separation method (NIPS), solvent evaporation method, in-situ growth method and layer deposition method, for the preparation of metal-organic framework (MOF) based membranes.

NIPS is commonly used in the industrial preparation of polymeric microfiltration (MF) and ultrafiltration (UF) membranes, which can only remove substances with size larger than 100 nm, such as colloidal particles, bacteria, viruses, and humus. Usually, small molecular organic substances such as dyes and racemates cannot be separated by NIPS membranes due to their relatively large pore size. NIPS MMMs with MOFs have been studied, yet MOFs were merely used as a filler and the MOFs loading was generally less than 5%.^1-9^ The increase of MOF loading will result in brittle membrane. As shown in Fig. C1, we have prepared NIPS MMM with MOF loading of 86%, in which the agglomeration of MOF particles causes a large number of cracks on the membrane surface and seriously affect the separation performance.

**Fig. C1** The photo of NH_2_-UiO-66 PVDF MMM-86% prepared by NIPS method.

The solvent evaporation method is usually utilized to fabricate dense MMMs, which is commonly used in the process with low flux (i.e., solvent separation or purification and separation of small molecules). Despite the MOF loading up to ~60%^19^, the permeation is still limited due to the dense packing of polymers resulted dissolution-diffusion separation mechanism. Similar to the NIPS method, increasing of MOF loading also leads to poor mechanical properties (Fig. C2).

**Fig. C2** The photo of NH_2_-UiO-66 PVDF MMM prepared by solvent evaporation method, the membrane was supported on an aluminum foil.

In situ growth is explored to prepare nanofiltration membranes, which utilizes the ordered pores in MOFs to reject small molecular dyes and allow the solvent molecule permeation. However, it is challenging to prepare large-area and continuous defect-free MOF separation layers, which makes it difficult to be applied in industrial applications. In addition, some studies have grown or deposited MOFs on the surface of porous substrates, where the MOFs acted as membrane adsorbent skin layer to separate dye molecules.^33-35^ Nevertheless, the interaction between the MOF particles as well as the MOF particles and the substrate is considerably weak. In such cases, the MOF particles can easily fall off because of a lacking adhesion, leading to the difficulties in the performing of separation process in cross-flow filtration mode. Therefore, although large permeate flux can be achieved, these membranes can only treat dye solution with low concentration. The pulverization problem and limited adsorption capacity greatly hinder its further application.

By combining the well-established TIPS method with the HoP method, we successfully weaved an ultra-high molecular weight polymer and 10 representative MOFs into flexible mixed matrix membranes. Thanks to the ultra-high molecular weight of polymers, the MOF loading can achieve 92% in the MMMs with good flexibility. This was previously inaccessible by using other membrane forming methods due to the polymer insolubility. Since the ultrahigh MOF loading makes it possible to access the merits of high permeation flux, efficient uptake or separation of small molecules (i.e., dyes, proteins and racemates), serving as an ideal complement to current microfiltration and ultrafiltration membrane technology. The obtained MMMs are chemically stable and can withstand a complex water environment. Moreover, this TIPS-HoP method is versatile and applicable to many porous solids in continuously preparing large-area separation membranes, and this research can help to move MOFs into industrial relevance.

The advantages and disadvantages of membrane fabrication methods are summarized in the following table. We have included all the above results, comparisons and related discussions in the revised manuscript (Page 2, Line 27-28; Page 3, Line 1-5; Page 3, Line 21-30; Page 4, Line 1-2) and supporting information (Supplementary Fig. 4).

| Method  **Table C1.** The summary of MOF-based membrane fabrication methods. | Polymer | Membrane process | MOF loading | Mechanism | Performance | Advantages | Limitations | Ref |
| --- | --- | --- | --- | --- | --- | --- | --- | --- |
| Nonsolvent induced phase separation | PAN | Microfiltration Ultrafiltration | $\leq5\%$ | Molecular sieving | The permeance for colloidal particles, proteins, bacteria, virus or humus removal can be  $>500$ L m^–2^ h^–1^ bar^–1^. | Easy fabrication, scalable | Cannot separate small molecules, low MOF loading | 1-9 |
|  | PVDF |  |  |  |  |  |  |  |
|  | PES |  |  |  |  |  |  |  |
|  | PSF |  |  |  |  |  |  |  |
| Solvent evaporation method | PEBA | Pervaporation | $\leq58.7\%$ | Solution-diffusion | The permeance for small molecules removal can be  $<1 L$ m^–2^ h^–1^ bar^–1^. Normally used in organic solvent purification, | Easy fabrication, scalable | Low permeate flux, limited MOF loading | 10-20 |
|  | PDMS |  |  |  |  |  |  |  |
|  | Matrimid® |  |  |  |  |  |  |  |
| In-situ growth method | AAO | Nanofiltration | Continuous layer | Molecular sieving and dielectric exclusion | The permeance for organic dyes or divalent ions removal can be  $<10 L$ m^–2^ h^–1^ bar^–1^ | Designable and tunable pore size, high selectivity | Difficult for fabricating large-area defect-free MOF separation layers | 21-32 |
|  | Polymer substrates |  |  |  |  |  |  |  |
|  | Ceramic tube |  |  |  |  |  |  |  |
| Layer deposition | Polymer and Inorganic substrates | Membrane adsorber | Continuous layer | Adsorption | The permeance for dyes removal can be  $2000 L$ m^–2^ h^–1^ bar^–1^ | High permeance | Low rejection for high concentration feed, difficult for large scale fabrication, pulverization problem | 33-35 |
| Thermally induced phase separation  (TIPS-HoP） | PE | Membrane adsorber | 92% | Adsorption,  molecular sieving | High permeance up to $125 L$ m^–2^ h^–1^ bar^–1^ for separation small molecules, can be used for separation of dyes, racemates and proteins | Good mechanical property and flexibility, easily large-scale, higher separation efficiency. | Selective separation based on adsorption rather than molecular sieving; | This work |
|  | PTFE |  |  |  |  |  |  |  |
|  | PP |  |  |  |  |  |  |  |

Polyacrylonitrile = PAN; poly(vinylidene fluoride) = PVDF; polyethersulfone = PES, polysulfone = PSF, poly(ether block amide) = PEBA; polydimethylsiloxane = PDMS; anodic aluminum oxide = AAO; polyethylene = PE; poly(tetrafluoroethylene) = PTFE; polypropylene = PP.

1. *J. Membrane. Sci.* **574**, 338 (2019); 2. *ACS Appl. Mater. Interfaces.* **9**, 21473 (2017); 3. *J. Membrane. Sci.* **569**, 48 (2019); 4. *J. Membrane. Sci.* **551**, 283 (2018); 5. *J. Membrane. Sci.* **492**, 392 (2015); 6. *Ind. Eng. Chem. Res.* **50**, 3798 (2011); 7. *J. Membrane. Sci.* **344**, 190 (2009); 8. *J. Membrane. Sci.* **526**, 355 (2017); 9. *J. Membrane. Sci.* **541**, 262 (2017); 10. *J. Membrane. Sci.* **544**, 68 (2017); 11. *J. Membrane. Sci.* **531**, 16 (2017); 12. *J. Membrane. Sci.* **582**, 307 (2019); 13. *J. Membrane. Sci.* **527**, 8 (2017); 14. *J. Membrane. Sci.* **549**, 217 (2018); 15. *J. Membrane. Sci.* **516**, 185 (2016); 16. *J. Membrane. Sci.* **502**, 48 (2016); 17. *J. Membrane. Sci.* **469**, 1 (2014); 18. *J. Membrane. Sci.* **577**, 129 (2019); 19. *J. Membrane. Sci.* **415**, 577 (2012); 20. *J. Membrane. Sci.* **579**, 309 (2019); 21. *ACS Appl. Mater. Interfaces.* **8**, 20459 (2016); 22. *ACS Appl. Mater. Interfaces.* **8**, 6236 (2016); 23. *AIChE J.* **62**, 538 (2016); 24. *Sci. Rep.* **4**, 4947 (2014); 25. *J. Mater. Chem. A.* **1**, 3688 (2013); 26. *J. Mater. Chem. A.* **5**, 15342 (2017); 27. *J. Membrane. Sci.* **523**, 561 (2017); 28. *ACS Appl. Mater. Interfaces.* **7**, 24082 (2015); 29. *ACS Appl. Mater. Interfaces.* **8**, 21979 (2016); 30. *Chem. Commun.* **51**, 918 (2015); 31. *Desalination* **385**, 75-82 (2016); 32. *ACS Appl. Mater. Interfaces.* **7**, 16157 (2015). 33. *Environ. Sci.: Nano* **6**, 1067 (2019); 34. *CrystEngComm* **20**, 5465 (2018); 35. *Environ. Sci.: Nano* **4**, 2205 (2017)

2. *The paper should be copyedited for clarity and understanding… will greatly increase the clarity of the work…*

Per suggestion, we have carefully checked the manuscript and revised accordingly.

3. *The lowest loading … there is no pure polymer membrane synthesized for control purposes. The authors should synthesize a pure polymer membrane…*

Per request, we have prepared a pure polymer membrane for comparison. The resultant membrane is dense and does not allow any solute or water to pass through.

We added the above discussions in the revised manuscript (Page 8, Line 12-14) and the supplementary Fig. 15.

4. *For the metal oxide controls (Figure S15), are the metal oxides in nanoparticle form? … need to be performed with particles on the same size range as the MOFs.*

The metal oxides used in the experiments are in their nanoparticle form. The particle sizes of ZnO and Al_2_O_3_ are 200 and 50 nm, respectively, which is in the same size range as the MOFs. To clarify this point, we added these details in the revised manuscript (Page 9, Line 11-14) and placed the SEM images of ZnO and Al_2_O_3_ in the revised supporting information (Page 2, 6-8 and Supplementary Fig. 18).

**Fig. C3** The SEM images of ZnO and Al_2_O_3_.

5. *The reviewers propose the addition of thermogravimetric analysis data to assess the actual MOF and polymer content of the MMMs produced. … If paraffin remains trapped in the MMMs, this would change the overall content of the MMMs produced. If paraffin remains, it should produce a weight loss event distinct from those of the MOF and two polymers, and the actual MOF wt% of the materials (as opposed to the presumed wt%) could be determined, strengthening the results.*

Per suggestion, we tested the thermogravimetric analysis (TGA) of NH_2_-UiO-66 PE MMMs with different loadings (Fig. C4). The MOF loadings calculated from the TGA analyses are consistent with the theoretical values (Table C2), proving that the paraffin is completely removed from the MMMs.

**Fig. C4** TGA of NH_2_-UiO-66 PE MMMs with different loadings.

**Table C2.** The calculation results of MOF loadings in NH_2_-UiO-66 PE MMMs.

| MOF loadings (wt%) | Residual mass (%) | Theoretical loadings (%) | Calculated loadings from TGA (%) |
| --- | --- | --- | --- |
| 0 | 0 | 0 | 0 |
| 28 | 17.6 | 28 | 27.6 |
| 54 | 33.1 | 54 | 53.3 |
| 61 | 37.5 | 61 | 60.4 |
| 70 | 43.1 | 70 | 69.4 |
| 86 | 53.5 | 86 | 86.1 |
| 92 | 57.1 | 92 | 91.9 |
| NH_2_-UiO-66 | 62.1 | 100 | 100 |

We have now included the above results and discussions in the revised manuscript (Page 6, Line 1-5) and supplementary information (Page 7, 19-20 and Supplementary Fig. 1 and Table 1).

6. *The reviewers suggest the authors change the naming scheme of their materials…*

Per request, we changed the name to mixed matrix membranes in the revised manuscript.

7. *Supplementary Figure 1: The depicted stress-strain curves of representative MOF/PE MMMs relative to PVDF and PAN alternatives seems to be incomplete… the lower-MOF content PE MMMs were not assessed…*

Per suggestion, we measured the stress-strain curves of NH_2_-UiO-66 PE MMMs with different MOF loadings (Fig. C5). The mechanical performance indeed decreases with the increasing of MOF loadings, but NH_2_-UiO-66 PE MMM-86% still possesses the flexibility and can bear bending without the formation of cracks. In comparison, The NH_2_-UiO-66 PVDF MMM-86% and NH_2_-UiO-66 PAN MMM-86% prepared by NIPS methods are very easily broken upon bending or stretching.

We have included the above results in the revised manuscript (Page 6, Line 11-19) and the supporting information (Supplementary Fig. 3,4).

**Fig. C5** The stress-strain curves of NH_2_-UiO-66 PE MMMs with different loadings, NH_2_-UiO-66 PVDF MMM-86% and NH_2_-UiO-66 PAN MMM-86%.

8. *Supplementary Figure 2, 3, 5, 6, 9, 13, 15: Please increase the size of the SEM images provided… Please increase the size/resolution of the PXRD traces provided ...*

Per suggestion, we have enlarged these figures in the revised supporting information.

9. *Please cite previous literature on UiO-66-NH_2_ and MIL-101 (Cr)’s ability to separate anionic and cationic dyes…it would also be enlightening to compare the performance of the MOFs alone vs. the MMMs- is any performance gained within the MMM form that can’t be accessed in the MOF alone? …*

We have cited the literatures (*J. Hazard. Mater.* **181**, 535 (2010); *J. Colloid. Interface. Sci.* **539**, 76 (2019); *Chem. Eur. J.* **22**, 10565 (2016)) related to MOF based dye separation and their charge-selective nature in the original manuscript. Per suggestion, we have discussed the relevant progresses in the revised manuscript (Page 9, Line 23-28).

Despite the fact that MOF crystalline powders have the ability to separate dye molecules based on adsorption, they are brittle in nature and can easily break down into fine powders. In industrial applications, these tiny particles will clog the pipes during separation and severe material loss will inevitably occur when flushing with fluid. Therefore, the fabrication of MOFs into granules or membranes is required. However, high MOF loading membranes with appropriate mechanical performance are hard to achieve by the reported method as stated previously. Our TIPS-HoP methodology offers a way to fabricate high-performance MMM with ultrahigh MOF loading irrespective of the MOF types and exhibits great potential in large-scale fabrication and separations.

To further prove this point, we filled columns by using NH_2_-UiO-66 powders (0.1 g, the same MOF amount in a 3.14 cm^2^ NH_2_-UiO-66 PE MMM-86%) for fuchsine acid (FA) separation (100 ppm). In the column with the same area (3.14 cm^2^) of MMMs used for separation (Fig. C6), the flux is high but cannot reject dye molecule in such high concentration. In the column with a diameter of 6 mm, the resistance is too high to allow the permeation of the solvent (Fig. C6) even under a pressure of 0.1 MPa. The column is clogged by the nanosized MOF particles.

**Fig. C6** The apparatuses for column filtration. NH_2_-UiO-66 in the column with the same area (3.14 cm^2^) of MMMs used for separation FA (left), NH_2_-UiO-66 in the column with a diameter of 6 mm used for separation FA (right).

We included the above discussions in the revised manuscript (Page 2, Line 14-21; Page 11, Line 1-18) and supporting information (Supplementary Fig. 24).

10. *We ask the authors to use care in reporting permeance vs. permeability... comment on the validity of the comparison to reported membranes… Please also include a table of the values with corresponding citations…*

We have corrected the word “permeability” to “permeance” in the revised manuscript. Since the permeance is directly related to the amount of wastewater that can be treated as well as energy consumption, the permeance of the membrane is more concerned in the practical application.

In addition, we have summarized the values listed in Fig. 3d with corresponding citations in the table below (Supplementary Table 6 in the revised supporting information).

**Table C3.** The comparison of separation performance

| Membrane | Permeance (L m^–2^ h^–1^ bar^–1^) | Molecular weight  cut off (Da) | Rejection (%) | Ref. |
| --- | --- | --- | --- | --- |
| NTR-7450 | 12 | Raffinose (504.4) | 60% | *Sep. Purif. Technol.* **14**, 155 (1998) |
| NF-PES-010 | 10 | Raffinose (504.4) | 90% | *J. Membrane. Sci.* **279**, 311 (2006) |
| NF-270 | 10.5 | Tryptophane (204.2) | 99% | *J. Membrane. Sci.* **279**, 311 (2006) |
| N30F | 11.1 | Fasavin CA 73 (742 - 1072) | 76.9% | *Ind. Eng. Chem. Res.* **44**, 7652 (2005) |
| Desal-HL-51 | 9 | Raffinose (504.4) | 90% | *J. Membrane. Sci.* **279**, 311 (2006) |
| Sepro NF 6 | 12.6 | Direct red 80 (1373) | 99.9% | *J. Membrane. Sci.* **493**, 690 (2015) |
| PEI/LDH | 19.8 | Methylene blue (319.8) | 97.9% | *J. Hazard. Mater.* **366**, 659 (2018) |
| ZIF- 8/PSS | 25.6 | Methyl blue (799.8) | 98.6% | *Angew Chem Int Ed Engl* **53**, 9775 (2014) |
| PEI/GA | 25.5 | Methyl blue (799.8) | 97.3% | *J. Membrane. Sci.* **524**, 214 (2017) |
| SG@GO/ ceramic | 33 | Eriochrome black T (461.3) | 98.3% | *J. Membrane. Sci.* **527**, 43 (2017) |
| Glycine-LDH | 56.6 | Eriochrome Black T (461.3) | 98.5% | *J． Mater. Chem. A.* **6**, 17148 (2018). |
| BCP membranes | 65 | Methyl blue (799.8) | 99.9% | *Nat. Commun.* **9**, 2294 (2018). |
| Multi-layered GO | 71 | Methyl blue (799.8) | 99.9% | *Nat. Commun.* **7**: 10891 (2016). |
| ZIF- 8/PEI | 78 | Congo red (696.6) | 99.2% | *J. Mater. Chem. A.* **5**, 15342 (2017) |
| COF-LZU1 | 76 | Congo red (696.6) | 99.2% | *Angew. Chem. Int. Ed.* **57**, 4083 (2018). |
| S-rGO membrane | 85.4 | Methylene blue (319.8) | 98.6% | *Adv. Mater.* **28**, 8669 (2016) |
| NSC-GO | 80 | EB (394.3) | 85% | *Nat. Commun.* **4**, 2979 (2013). |
| GO-IPDI | 83.3 | Methyl blue (799.8) | 99.9% | *Chem. Eng. J.* **322**, 657 (2017) |
| TMC-GO | 30 | Rhodamine-WT (566.9) | 99.9% | *Environ. Sci. Technol.* **47**, 3715 (2013). |
| NH_2_-UiO-66-MIL-100(Cr)-PE MMM-86% | 117.8 | Crystal violet (407.9) | 99.2% | This work |
|  | 125.7 | Methylene Blue (319.8) | 99.0% |  |
|  | 111.2 | Rhodamine B (479) | 98.8% |  |
|  | 103 | Orange G (452.3) | 99.6% |  |
|  | 116.8 | Congo Red (696.6) | 99.1% |  |
|  | 109.8 | Fuchsine acid (585.5) | 98.9% |  |
|  | 105 | Rose bengal (1017.6) | 99.2% |  |

11. *Supplementary Table 4:… ee values are reported in Supplementary Table 4, but no comparison is given in terms of permeance. Please include those values in the table, elaborate on this point in the text…*

We have compared the *ee* value, solvent permeance, and solvate permeance with the reported works in the table below and added it into the revised supporting information (Supplementary Table 7). We also included the discussion of this comparison in the revised manuscript (Page 13, Line 1-14).

| MOFs | Substrate | Method | Enantiomeric excess  (ee%) | Separation method | Flux for solute  (mmol m^–2^ h^–1^) | Flux for solvent  (L m^–2^ h^–1^ bar^–1^) | Ref |
| --- | --- | --- | --- | --- | --- | --- | --- |
| Ni_2_(L-asp)_2_(bipy) | Nickel net | Single nickel source | R-MPD/S-MPD (32.5%) | Filtration | N/A | 53.3 | 29 |
| L-His-ZIF-8 | AAO | Secondary growth | R/S-1-phenylethanol (76%) | Static diffusion | 5.11 | N/A | 30 |
| [Zn_2_(bdc)(L-lac)(dmf)] | Porous ZnO | Secondary growth | R-MPS/S-MPS (33%) | Static diffusion | 0.15 | N/A | 31 |
| Ni_2_(L-asp)_2_(bipy) | α-Al_2_O_3_ disks | Secondary growth | R-MPD/S-MPD (35.5%) | Static diffusion | 1 | N/A | 32 |
| Zn_2_(cam)_2_(dabco) | QCM substrate | Layer by layer | R-HDO/S-HDO (21.6%) | Static diffusion | N/A | N/A | 33 |
| [Zn_2_(bdc)(L-lac)(dmf)] | PE | MMMs | R-MPS/S-MPS (74%) | Filtration | 11.5 | 85 | This work |

**Table C4.** The comparison of separation performance.

**Referee 2:**

1. *The results claimed in the abstract are impressive with “flux higher than 1257 L m^–2^ h^–1^ MPa^–1^ and 99 % rejection of small organic dyes (as low as 319 Da)”. … please supply in the supplementary information a table listing permeance (L m^–2^ h^–1^ MPa^–1^) clearly linked to the rejection of each dye used for each MOF/polymer combination. Please add the error bars for these measurements.*

Per request, we have now listed the detailed data and added the error bar in the Supplementary Table 5.

**Table C5.** Summary of the dye separation performance of MOF PE MMMs.

| MOF PE MMMs-86% | Dyes | Concentration (ppm) | Molecular weight (Da) | Permeance  (L m^–2^ h^–1^ bar^–1^) | Rejection |
| --- | --- | --- | --- | --- | --- |
| NH_2_-UiO-66 | OG | 100 | 452.3 | 115.9 ± 2 | 99.1 ± 0.1 |
|  | CR | 100 | 696.6 | 102.7 ± 3 | 99 ± 0.05 |
|  | FA | 100 | 585.5 | 111.4 ± 2.5 | 99.1 ± 0.08 |
| MIL-100(Cr) | CV | 100 | 407.9 | 112.5 ± 1.5 | 99.2 ± 0.15 |
|  | RB | 100 | 479 | 108 ± 1.3 | 99.3 ± 0.1 |
|  | MB | 100 | 319.8 | 120 ± 1.6 | 99.0 ± 0.1 |
| Mixed NH_2_-UiO-66 with  MIL-100(Cr) | CR | 100 | 696.6 | 116.8 ±1.8 | 99.1 ± 0.1 |
|  | FA | 100 | 585.5 | 109.8 ± 2.6 | 98.9 ± 0.15 |
|  | CV | 100 | 407.9 | 117.8 ± 2 | 99.2 ± 0.08 |
|  | RB | 100 | 479 | 111.2 ± 2.5 | 98.8 ± 0.08 |
|  | MB | 100 | 319.8 | 125.7 ± 1.5 | 99. 0 ± 0.1 |
|  | OG | 100 | 452.3 | 103 ± 1.5 | 99.2 ± 0.05 |

2. *Please note that “permeance” is the most appropriate term. Use preferentially unities with bar, which are the most used in the field and would be easier to directly compare.*

Per suggestion, we have changed “permeability” to “permeance”. We also changed unities with the “bar” in the revised manuscript.

3. *To avoid having adsorption as cause of an apparent rejection, please report also the concentration values of the feed, retentate and permeate for the performance measurements. UHMWPE gives a good mechanical property but the extrusion might be challenging, the melt viscosity is high and the interaction with the MOFs could be poor. Please provide microscopy images at higher magnification, detailing the interface between the MOF crystals and the polymer.*

The total amount of the feed is 2 L and its concentration is 100 ppm. Around 70 mL h^–1^ water passed through the membrane with an area of 3.14 cm^2^ and the feed was resupplied by fresh solution (100 ppm) every hour, thus, the feed concentration remained almost constant during the separation tests. The permeance and rejection values are listed in Supplementary Table 5. As discussed in our original manuscript, the micron-sized channels between the MOF particles translate into fast water permeation, while the nano-sized cavities with designable pore environment in the MOFs lead to selective rejection of solutes. The ultrahigh MOF loading assures high rejection of dye molecules in relatively high concentration within a long period.

We have discussed the effect of high-density PE (HDPE) and ultra-high molecular weight PE on viscosity. Ultra-high molecular weight PE (UHMWPE) is very sticky and difficult to be extruded. Therefore, we added paraffin oil as a flow agent and mixed HDPE and UHMWPE to a certain ratio to guarantee the successful melt-processing of MMMs. SEM images of NH_2_-UiO-66 PE MMM-86% at higher magnification is shown in the figure below.

**Fig. C7** Top-view (left) and cross-section SEM images (right) of the NH_2_-UiO-66 PE MMM-86%.

Accordingly, we have added the above discussions and results in the revised manuscript (Fig. 2d) and supporting information. (Page 8, Line 3-7; Supplementary Fig.6)

**Referee 3:**

1. *The membranes reported in this work are membrane adsorbers or membrane sorbents... the authors did not cite any literature on membrane adsorbers or membrane chromatography.…The working principles of membrane absorbents are different … In mixed matrix membranes, the MOF-based fillers are expected to facilitate the transport of molecules (although the mechanism can be complex)...the adsorption of species on surfaces and pores of MOFs becomes dominant, and regeneration of the chemicals adsorbed in the sorbents. … Recent studies have shown that MOF membrane adsorbers can efficiently adsorb and remove dye molecules and give high water permeance of 20,000 L m^-2^ bar^-1^ h^-1^, which is much higher than the water permeance achieved in this study (120 L m^-2^ bar^-1^ h^-1^)….*

In the revised manuscript, we have thoroughly reviewed the characteristics and advantages of this TIPS-HoP technique compared with the reported mixed matrix membrane (MMM) fabrication methods. We have summarized the pioneering works relevant to MOF membrane adsorbers and discussed the key challenges in the area. Indeed in the MOF membrane adsorbers reported, the researchers usually use dead-end cell filtration mode and much lower dye concentration (< 20 ppm) as reported in the works mentioned by the reviewer (*Environ. Sci-Nano.* **6**, 1067 (2019); *CrystEngComm* **20**, 5465 (2018); and *Environ. Sci-Nano.* **4**, 2205 (2017)). In addition, the interaction between the MOF and the substrate is weak and the MOF particles can easily fall off without adhesion of additional polymeric binders, which also leads to the difficulties in increasing the thickness of a continuous MOF membrane and performing separation process in cross-flow filtration mode. Therefore, although large permeate flux can be achieved, these membranes can only treat dye solution with low concentration. Further, the dead-end filtration mode suffers from severe concentration polarization problem and cannot be applied in industry to treat large amount of waste water with high pollutant concentration. The pulverization problem and limited adsorption capacity greatly hinder its further application.

Indeed, as we discussed in the original manuscript, the working principle of our MMMs with high-loadings of MOFs is based on fast and efficient adsorption, which is different from conventional MF or UF membranes. However, we performed the dye separation experiments in similar conditions used in MF and UF, for instance, cross-flow filtration mode, dye concentration (100 ppm), continues separation for hours, and recovery process. Since the MOF particles are interwoven by the polymer with ultrahigh molecular weight, there is no loss of MOF during operation. Therefore, we believe the flux and selectivity obtained in these experiments can be compared with other separation membranes. Furthermore, the ease of scale-up and assembling into membranes module demonstrates its great potential in real membrane applications.

To further clarify these points, we have included the above discussions in the revised manuscript (Page 2, Line 27-28; Page 3, Line 1-5).

2. *Certainly, there are some interesting results reported in this work. The authors report a new method… The authors evaluated a series of MOFs and dye molecules…It is not clear whether the molecules are adsorbed on the surface or within the micropores and how the pore structure of MOFs determine the separation performance.*

The pore size as well as zeta potential of the MOFs determine the separation performance. The pore size of NH_2_-UiO-66 and MIL-100(Cr) are 1.1 nm and 1.6 nm, respectively, and they can efficiently adsorb dye molecules with opposite charge and suitable molecular sizes. Dye molecules with opposite charge but larger sizes cannot be adsorbed by relevant MOFs. For example, since the size of RB (1.56 × 1.35) is bigger than NH_2_-UiO-66 pore size, there is no rejection for RB filtrated by NH_2_-UiO-66 PE MMM-86%.

To further confirm where the molecules are adsorbed, we compared the BET surface area, pore size distribution and morphologies of the membranes before and after filtration. The BET surface area of the NH_2_-UiO-66 PE MMM-86% after 5-h CR removal test decreases from 746 to 253 m^2^ g^–1^ and can be easily regenerated to 80% of its original porosity after washing. As shown in the pore size distributions profiles (Supplementary Fig. 16), the pore volume contributed by micropores are significantly decrease after CR removal, revealing that the micropores inside MOFs are partially filled by dye molecules. The SEM images show that the dye adsorption also occurred on the surface (Supplementary Fig. 17). To further confirm the function of the hierarchical porous structure for CR removal, we fabricated nonporous Al_2_O_3_- and ZnO-mixed membranes (the particle sizes of ZnO and Al_2_O_3_ are 200 and 50 nm, respectively) using identical TIPS-HoP method as comparisons (Supplementary Fig. 18). These two membranes exhibit extremely low water flux and almost no CR rejection ability.

**Table C6** The molecular sizes of dyes

|  | Crystal violet (CV) | Methylene Blue (MB) | Rhodamine B (RB) | Orange G  (OG) | Congo Red  (CR) | Fuchsine acid (FA) |
| --- | --- | --- | --- | --- | --- | --- |
| Size | 1.42 × 1.01 | 1.38 × 0.64 | 1.56 × 1.35 | 1.62 × 0.94 | 2.61 ×0. 86 | 1.25 × 1.07 |

To further clarify of this point, we rephrased the corresponding paragraph in the revised manuscript (Page 9, Line 8-14, 20-22; Page 10, Line 27-30).

3. *The authors need to compare the separation efficiency with conventional adsorbents. For example, if the same polymer supported MOF particles are packed into one column and the dye solutions are purged through the column, what is the separation performance?*

If we only use MOF to pack the column, the permeance and rejection depend on the packing density MOF particles. High density will lead to low permeance, while low density will result in low rejection. The HDPE is in granule form with a diameter of 3.7 mm and a thickness of 1.6 mm. When we packed the same amount of 0.086 g NH_2_-UiO-66 mixed with 0.0112 g HDPE and 0.0028 g UHMWPE to fill a column (i.d. 6 mm) to perform fuchsine acid (FA) separation (100 ppm) under 0.1 MPa, the result is the same with pure MOF powder due to the very low proportion of polymer.

Then we tried to use quartz sand to disperse MOF particles (0.1g, the same amount of a MMM with an area of 3.14 cm^2^) under atmospheric pressure, the rejection for FA (100 ppm) was calculated to be 47.7%.

Moreover, as we discussed above (the response to Referee 3, Q1), the conventional adsorbents suffer from pulverization problem and limited adsorption capacity, as well as that the dead-end filtration facing severe concentration polarization problem and cannot be applied in industry for large-amount water treatment.

The above results and discussions are included in the revised manuscript (Page 11, Line 1-18) and supporting information (Supplementary Fig. 24, 25 ).

**Fig.** **C8** The apparatus for dyes filtration by using quartz sand-dispersed MOF particles under atmospheric pressure (left), the UV-vis of 100 ppm feed FA and after column filtration (right).

4. *Rejection is not a proper parameter to characterize the purification of dye molecules in membrane adsorber because it strongly depends on the feed concentration. The adsorption capacity of dye molecules in MOFs is more important. The authors used a variety of MOF materials but there is not a clear connection between the MOF structures and the adsorption performance. The dye molecules may be adsorbed on the surface of the MOFs...*

Indeed, the membrane adsorber strongly depends on the feed concentration. As we discussed above, previously reported MOF membrane adsorbers can only treat a small amount of dye polluted water with a low concentration (< 20 ppm). However, in this work, we performed the dye separation experiments in similar conditions used in MF and UF, for instance, cross-flow filtration mode, dye concentration (100 ppm), continues separation for hours, and recovery process. Despite the separation mechanism is different from MF and UF membranes, we believe the flux and rejection obtained in these experiments can be compared with other separation membranes.

As we discussed in the original manuscript, we have thoroughly investigated the connection between MOF structure and adsorption performance. We measured the dyes adsorption capacity and kinetic adsorption of different MOFs and found it is directly related to the membrane separation performance. The electrostatic attraction between the MOFs and dye molecules determines the dye separation performance. Through the specific surface area analyses, pore size distribution calculations and the SEM images of the pristine, fouled and cleaned of NH_2_-UiO-66 MOF PE MMM-86% (Supplementary Fig. 16, 17), we confirmed that the dye molecules are adsorbed inside of the MOF pores and on the surface of MMMs. The charge type of the membrane and the pore structure of the MOF mainly affect the adsorption of the dye, which is in consistent with the previous work (Ref. 28, 40 - 42).

5. *For the chiral and protein separations in the dead end cells, were these adsorption or dynamic separation? The chiral separation data are not supported by evidence. Whether the membrane adsorber is intrinsically chiral … The authors only provided one enantiomeric excess value … What was the enantiomer permeance or flux? What about the other membranes without chirality…*

The chiral and protein separations are performed in a dynamic process. Zn-BLD is a chiral MOF and its separation mechanism is discussed in the previous work (Ref. 38, 43). In this manuscript, we focused on the incorporating of this chiral MOF in the MMMs and demonstrated their superior chiral separation performance.

Per request, we further conducted cycled chiral separation tests. We collected permeate after 15-min dynamic separation and then cleaned the membrane. The separation-washing process was performed for 5 cycles. The results showed that the *ee*% values maintained around 74% after 5 times, and the acetonitrile permeance was kept at 85 L m^–2^ h^–1^ bar^–1^ (Fig. C9). The enantiomer permeance of R-MPS is calculated to be 11.5 ± 0.5 mmol m^–2^ h^–1^ (Fig. C10), This ee% value is among the best for the membrane-based chiral separations and enantiomer permeance is hardly achieved by the reported membranes with similar separation ee% value (Supplementary Table 7).

The *ee*% value by using NH_2_-UiO-66 PE MMM-86% for separation is close to zero, revealing that the membrane without chirality cannot be used to separate enantiomers (Fig. C11).

Accordingly, we included the above discussions in the revised manuscript (Page 13, Line 1-14) and supporting information. (Supplementary Fig. 26, 28, 29)

**Fig. C9** The separation performance of Zn-BLD PE MMM-86% for chiral MPS (concentration, 0.1 mg mL^–1^; applied pressure, 0.05 MPa).

**Fig. C10** The separation performance of Zn-BLD PE MMM-86% for R/S-MPS (concentration, 0.1 mg mL^–1^; applied pressure, 0.05 MPa).

**Fig. C11** High-performance liquid chromatogram of chiral MPS permeate after filtration by NH_2_-UiO-66 PE MMM-86%.

6. *Only one selective factor was derived from the protein concentration profiles. The authors attributed to the separation to electrostatic attraction without fundamental understandings.*

The protein separation is mainly due to electrostatic interaction, which has been investigated and demonstrated in the ref. 47-49 in the original manuscript. In these reported works, the researchers mainly focused on the selective factor. We employed this well-established separation principle to demonstrate the advantages of MMMs, with higher selectivity and potentials in a real application.

To clarify this point, we included the above discussions in the revised manuscript (Page 13, Line 15-16).

7. *The authors claimed that “and this roll-to-roll production can be easily scaled up by retrofitting in current industrial facilities and processes”. No evidence was provided – diagram shows roll to roll, but it is not clear whether the authors really performed this in reality. The size of 15 cm^2^ x 13 cm^2^ is quite small for roll-to-roll manufacturing. From the supplementary information it seems that only single sheets were made. A detailed description of the equipment used for the roll to roll process, the sizes of membranes produced, is required. The authors did not mention the thickness of the membranes produced.*

We used a homemade apparatus to fabricate the membrane, and an MMM with a size of 90 cm × 10 cm prepared by TIPS-HoP method is demonstrated in the figure below. We are currently applying a patent for the apparatus, and the detailed device information and related parameters are beyond the scope of this manuscript. The thickness used in the separation tests is around 350 μm.

**Fig. C12** Photograph of ZIF-8 PE MMMs-86% with an area of 90 cm × 10 cm.

Accordingly, we added the above figure in the revised supporting information (Supplementary Fig. 2).

8. *Diagrams of equipment in Supplementary Fig 11 are inadequate – no dimensions or volumes provided. How is pressure maintained in the crossflow cell? No backpressure valve reported.*

The area of effective filtration cell is 3.14 cm^2^, and the total amount of the feed is 2L. The pressure is adjusted and maintained by a valve. We redrew the schematic of the apparatus for dyes filtration and added it to the revised supporting information (Supplementary Fig. 14).

**Fig. C13** Schematic of the apparatus for dyes, racemates and proteins filtration, (a) cross-flow filtration system and (b) dead-end filtration system

9. English needs to be improved …

We have carefully checked the manuscript and corrected some typos accordingly.

Reviewers' comments:

Reviewer #2 (Remarks to the Author):

The authors revised the paper in detail. The manuscript is now ready to publish.

Reviewer #3 (Remarks to the Author):

The authors have made efforts and performed extensive amount of work to address comments raised by this reviewer and other reviewers. As pointed out by this reviewer and other reviewers, the manufacturing method is novel and the revised manuscript further emphasised the method rather than the separation performance. This will advance the manufacturing of MOFs into industrially useful membrane products. The quality of the work has been improved. The authors also satisfactorily addressed most questions. However, this reviewer still some concerns about the claims and request the authors to provide more explanation and clarification. It is disappointing that the authors selectively showed reviewer comments in their response letter and ignored some comments and questions raised by the reviewers. This makes it difficult to evaluate whether the authors have addressed all the reviewer comments (including those raised by other reviewers)

One major concern is that the authors did not clearly explain the working principles of their membranes as membrane adsorbers or membrane chromatography (the concepts already exist and have been widely used). As raised in the original comments, this reviewer expressed concerns about the difference of membrane adsorbers and mixed matrix membranes. The composite membranes reported in this work consist of high-loading MOFs supported by high molecular weight polymers. Indeed, the membranes combine the superior adsorption properties of MOFs and excellent processability and flexibility of polymers, and to some extent can be considered as mixed matrix membranes, as pointed out by Reviewer 1. However, strictly speaking, the resulting membranes are still membrane adsorbents because they require periodical desorption and regeneration to recover the capability for separation of small molecules. This is different from classical nanofiltration membranes or other MMMs that allow continuous
separation without desorption process. For all the molecules adsorbed in the membranes, water and solvent would be required to wash the membranes and the recovery of molecules (e.g. chiral or protein) from the washed solvent would require additional separation process and energy input. Therefore, the authors cannot simply claim that their performance are superior to other separation membranes. This reviewer requests the authors to add one paragraph to discuss the difference of their membranes and point out the advantages and disadvantages of their membranes as new generation of membrane adsorbers. This is important to justify the novelty of this work. The authors also need to add explanation of the desorption efficiency of their membranes.
Minor comments
The authors need to check the accuracy of their methods. In Fig. S28, the operation pressure is 0.05 MPa, this is below atmospheric pressure?
The stress strain curves do not show all the data clearly. The authors should present a table to summarize the data. 
The authors did not address the question on the change of enantiomeric excess as a function of time. Was the EE value obtained from the average vale of the 15-min dynamic separation? Only one HPLC curve was provided. At least, the authors should provide the curves of racemic mixtures.

**Responses to referees’ comments**

**Referee 3:**

1. *…However, strictly speaking, the resulting membranes are still membrane adsorbents because they require periodical desorption and regeneration to recover the capability for separation of small molecules. This is different from classical nanofiltration membranes or other MMMs that allow continuous separation without desorption process. For all the molecules adsorbed in the membranes, water and solvent would be required to wash the membranes and the recovery of molecules (e.g. chiral or protein) from the washed solvent would require additional separation process and energy input. Therefore, the authors cannot simply claim that their performance are superior to other separation membranes. This reviewer requests the authors to add one paragraph to discuss the difference of their membranes and point out the advantages and disadvantages of their membranes as new generation of membrane adsorbers. This is important to justify the novelty of this work. The authors also need to add explanation of the desorption efficiency of their membranes.*

As indicated by the reviewer and also elaborated in the previously revised manuscript, the MOF membrane separation process, unlike traditional nanofiltration, is mostly based on adsorption mechanism. Therefore the membranes prepared in this work did require periodical desorption and cleaning to recover the capability for separation of small molecules. However it is worth noting that the membrane cleaning is indeed a very common and standard routine operation procedure in many separation processes, especially nanofiltration and reverse osmosis, since the fouling would lead to a sharp decrease of permeate flux.

Per request, in this revised manuscript, we further summarized representative works related to the fouling and regeneration process of nanofiltration membranes and other MMMs (Table C1). It can be seen that the antifouling and recovery performance of our MOF MMMs is actually superior to those of the reported filtration membranes. Importantly no additional energy input is required during the MOF membrane separation process. Specifically we performed the dye separation experiments in comparable conditions commonly used in microfiltration and ultrafiltration (i.e. cross-flow filtration mode, dye concentration (100 ppm), continues separation for hours and recovery process), higher water flux can be achieved by these ultrahigh MOF loading membranes.

**Table C1** The summary of representative works related to the fouling and regeneration process of nanofiltration membranes and other MMMs.

| Membrane | Permeate flux  （L m^–2^ h^–1^ bar^–1^） | Time interval / Flux attenuation ratio | Flux recovery ratio | Ref |
| --- | --- | --- | --- | --- |
| SiO_2_-PSS | 26.9 | 2 h / 60% | 93% | 1 |
| CS-MMT | 15.6 | 2 h / 60% | 91% | 2 |
| QPEI/PES | 12.5 | 1 h / 40% | 94% | 3 |
| Fe-phos-(PEI) | 11.76 | 5 h / 30% | 70% | 4 |
| TiO_2_-HMDI | 30.5 | 1 h / 64% | 97% | 5 |
| PSF/GO | 50 | 3 h / 10% | 93% | 6 |
| GO/MoS_2_ | 12 | 0.5 h / 60% | 83.4% | 7 |
| VES/AgCl–PEI | 10 | 1 h / 35% | 74% | 8 |
| M-80COOH | 30 | 1 h / 40% | 70% | 9 |
| PDA/PEI | 24 | 3 h / 50% | 90% | 10 |
| NH_2_-UiO-66 PE MMMs | 100 | 5h / 10% | 97% | This work |

*1. Sep. Purif. Technol.* **2015,** *146*, 50-59; 2. *Chem. Eng. J.* **2015,** *265*, 184-193; 3. *Acs Sustain. Chem. Eng.* **2015,** *3*, 690-701; 4. *J. Membrane. Sci.* **2018,** *555*, 56-68; 5. *Sep. Purif. Technol.* **2019,** *218*, 8-19; 6. *Chem. Eng. J.* **2019,** *363*, 33-42; 7. *J. Membrane. Sci.* **2019,** *574*, 112-123; 8. *J. Membrane. Sci.* **2018,** *566*, 168-180; 9. *J. Membrane. Sci.* **2018,** *563*, 655-663; 10. *Chem. Eng. J.* **2018,** *341*, 371-382.

The membranes reported to date that can realize efficient chiral or protein separation are often based on selective adsorption mechanism rather than size-sieve mechanism. Recovery of chiral compounds or proteins from the washed solvent are needed. Similarly, better performance achieved using these ultrahigh MOF loading membranes prepared in this work provides a chance to minimize energy consumption.

In the original manuscript, flux recover ratio was used to illustrate the desorption efficiency of our membranes. To clarify this point, in this revised manuscript, we further compared this parameter with the literature reports pertaining to membrane regeneration.

Beyond the advantages these new MOF membranes had showed, we also realize that further optimization and study are needed to improve the selectivity and flux especially under complicated water pollution systems (organic, inorganic, bio, etc.). Also, the cost of production and energy consumption should also be further reduced and optimized in a practical water purification system. Nevertheless, we believe the current TIPS-HOP strategy is indeed a powerful method to fabricate MOF MMMs for highly efficient industrial separation processes.

Per suggestion, we have now included all the above discussions in the revised manuscript (page 12, line 6-28) and supporting information (Supplementary Table 10).

*Minor comments
1. The authors need to check the accuracy of their methods. In Fig. S28, the operation pressure is 0.05 MPa, this is below atmospheric pressure?*

Per suggestion, we corrected this description to “pressure difference, 0.05 MPa”.

*2. The stress strain curves do not show all the data clearly. The authors should present a table to summarize the data.*

Per request, we have now listed the detailed data in the Supplementary Table 2.

*3. The authors did not address the question on the change of enantiomeric excess as a function of time. Was the EE value obtained from the average value of the 15-min dynamic separation? Only one HPLC curve was provided. At least, the authors should provide the curves of racemic mixtures.*

The ee% value was decreased to 47.8% from 15 to 30 min, and will be further decreased with prolonged time. However, both ee% value and solvent permeance performance can be recovered by washing and also remained cross cycles.

The ee% value was obtained from the average value of the 15-min dynamic process. We collected all the permeate in the 15-min dynamic process and performs the ee% value tests.

Per suggestion, we now added the above discussions and all the corresponding HPLC curves in the revised manuscript (Page 11, line 11-14) and supporting information (Supplementary Figure 27).

Reviewers' comments:

Reviewer #3 (Remarks to the Author):

This paper is titled as an "ultrafast membrane". However after two reviews the authors finally agree that it is actually a membrane adsorber, and must undergo regeneration. For me, this is simply not made clear enough - all the way from the title onwards, the idea of a separation membrane and a membrane adsorber are confused. At least the term membrane adsorber should be used in the title.

While this is an interesting idea for making membranes, the mode of operation is not clearly explained.

**Responses to referees’ comments**

**Referee 3:**

1. *This paper is titled as an "ultrafast membrane". However after two reviews the authors finally agree that it is actually a membrane adsorber, and must undergo regeneration. For me, this is simply not made clear enough - all the way from the title onwards, the idea of a separation membrane and a membrane adsorber are confused. At least the term membrane adsorber should be used in the title. While this is an interesting idea for making membranes, the mode of operation is not clearly explained.*

Per request, we have added the phrase “membrane adsorber” and relative discussions throughout the manuscript starting from the title, abstract and introduction in the revised manuscript. For example, we added “membrane adsorbers are able to sufficiently increase the flow rate through anchoring functional groups onto the porous membranes (e.g., resins). However, they can only treat solutions with very low concentrations due to limited porosity and adsorption ability. Therefore, incorporation of porous and functional MOFs into membrane adsorbers with ultrahigh loading is highly desired” in the introduction part of the revised manuscript (Page 2, line 15-19). To make it clear, we also emphasized the operation mode in revised manuscript (Page 4, line 14-15, Page 13, line 13-14, 16), and the schematic of the apparatus for the experiments was presented in the original supporting information (Supplementary Figure 14).
